# 4-Hydroxy-6-alkyl-2-pyrones as nucleophilic coupling partners in Mitsunobu reactions and oxa-Michael additions

**DOI:** 10.3762/bjoc.10.116

**Published:** 2014-05-20

**Authors:** Michael J Burns, Thomas O Ronson, Richard J K Taylor, Ian J S Fairlamb

**Affiliations:** 1 Department of Chemistry, University of York, Heslington, York, YO10 5DD, U.K.

**Keywords:** heterocycles, Mitsunobu reaction, oxa-Michael addition, 2-pyrone, vinyl ethers

## Abstract

Two mild and efficient strategies have been developed for the *O*-functionalisation of 4-hydroxy-6-alkyl-2-pyrones, by using them as nucleophilic partners in oxa-Michael additions and the Mitsunobu reaction. The reactions proceed in moderate to excellent yields on a range of substrates containing useful functionality. The reactions serve as practical and valuable synthetic methods to construct complex 2-pyronyl ethers, which are found embedded in a number of natural products.

## Introduction

**Figure 1:**
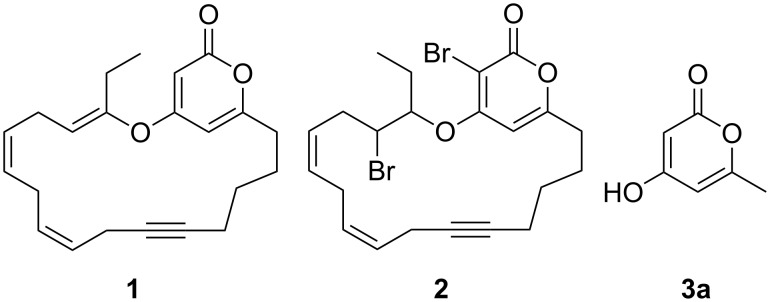
The phacelocarpus 2-pyrones **1** and **2**.

The 2-pyrone motif is a prevalent structural feature of many complex natural products and biologically active compounds [1–2]. Various functionalised 2-pyrones have been identified as promising candidates for the treatment of illnesses ranging from Alzheimer’s disease [3] to cancer [4]. An important sub-class of pyrones are the 4-hydroxy-2-pyrones, which are sometimes found embedded into larger natural products as pyronyl ethers, such as in the phacelocarpus 2-pyrones **1** and **2** (Figure 1). These compounds are secondary metabolites isolated from the Australian marine red alga *Phacelocarpus labillardieri* [5]; similar compounds from the same family have been shown to exhibit phospholipase A_2_ (PLA_2_) inhibitory activity [6]. Whilst the chemistry of 2-pyrones is generally well-developed [7], efficient routes to these types of complex structural units are elusive, and the total synthesis of these and similar natural products remains a challenge [8].

Simple 2-pyrones such as 4-hydroxy-6-methyl-2-pyrone (triacetic acid lactone, **3a**, Figure 1) are readily and cheaply available, making them seemingly ideal building blocks for the synthesis of such complex pyrone-containing molecules. As heterocyclic aromatic enols, they have a high acidity and dense functionality which leads to a diverse reactivity profile. This means that 4-hydroxy-2-pyrones are also useful precursors to a number of other structural units and versatile intermediates in organic synthesis.

**Scheme 1:**
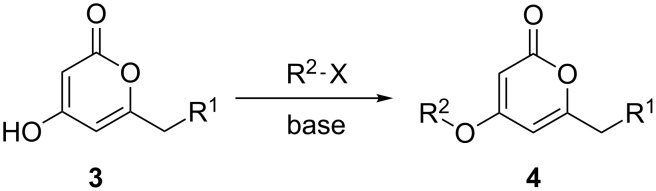
Generalised *O*-functionalisation of 6-alkyl-4-hydroxy-2-pyrones **3**.

Despite, or perhaps because of, this varied reactivity, *O*-functionalisation reactions of 4-hydroxy-2-pyrones, to afford 2-pyronyl ethers (e.g., Scheme 1), remain almost entirely limited to reactions with methylating agents or simple alkyl or acyl halides. These often require heating with a base such as K_2_CO_3_ or DBU, and the available functionality is therefore limited to esters or simple primary alkyl groups [9–11]. Even in simple cases the yields obtained are variable as the 2-pyrone unit is prone to degradation under harsh conditions, representing an interesting synthetic chemistry challenge to address. The ability to install more complex functionality on the hydroxy group of 6-alkyl-4-hydroxy-2-pyrones would be of considerable synthetic value.

The Mitsunobu reaction is a well-established, widely used and invaluable tool for synthetic chemists [12]. It is usually employed to couple an acidic nucleophile with a primary or secondary alcohol, and as a mild reaction it tolerates a range of functionality in both coupling partners, allowing it to be used on complex and sensitive substrates. Given the high acidity of hydroxypyrones such as **3** (Scheme 1; R^1^ = H, p*K*_a_ = 4.94 [13]), they would appear to be ideal coupling partners in the Mitsunobu reaction.

We recently published an example of the Mitsunobu reaction using the compound 4-hydroxy-6-methyl-2-pyrone (**3a**) [14]. To the best of our ability, we could find only limited precedent for this procedure being used previously in this way [15–17]. In our case, we were able to further modify the resulting pyronyl ether forming a trisubstituted enol ether, which then underwent a Suzuki–Miyaura cross-coupling or direct arylation-type reaction. As part of our extensive studies on reactions involving 2-pyrone derivatives, we report herein a significant expansion of the Mitsunobu protocol to a variety of different coupling partners, along with an alternative route to the formation of 2-pyronyl enol ethers using an oxa-Michael addition to propiolate esters. Both procedures are mild, tolerate a wide range of functionality, and afford good to excellent yields of products in the majority of cases.

## Results and Discussion

### Mitsunobu reactions

**Scheme 2:**
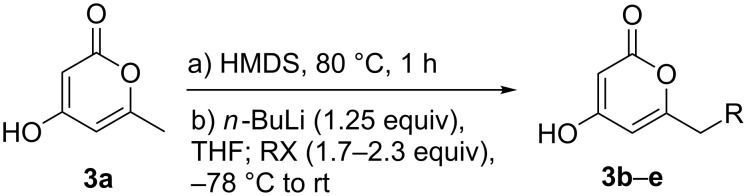
Synthesis of alkylated 2-pyrones **3b**–**e**.

Using the standard conditions of stirring diisopropyl azodicarboxylate (DIAD) and triphenylphosphine in dichloromethane at room temperature for 18 hours, we tested the substrate scope of the reaction (Table 1). In addition to the silyl-protected alcohol reported previously [14] (Table 1, entry 3), we found that a variety of useful functionality on the alcohol was well-tolerated, including a terminal alkene (Table 1, entry 2), a tosylate leaving group (Table 1, entry 4), and a halide (Table 1, entry 5). Somewhat more exotic functional groups such as a phosphonate ester (Table 1, entry 6) or a dimethyl acetal (Table 1, entry 7) were still tolerated in the reaction, albeit in more modest yields. A variety of alkylated 2-pyrones **3b**–**e** were synthesised according to the method of Hsung and co-workers [18], in order to further explore the scope of the Mitsunobu process. This involves a one-pot silyl-protection of the hydroxy group of 6-methyl-4-hydroxy-2-pyrone **3a** with HMDS, followed by lithiation and alkylation (Scheme 2).

**Table 1:** Synthesis of 2-pyronyl ethers **4a**–**l**.

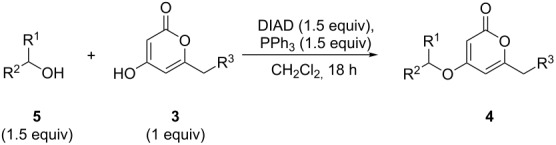				

Entry	Alcohol	Pyrone	Product	Yield (%)^a^

1	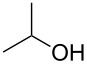 **5a**	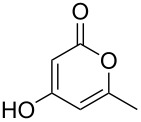 **3a**	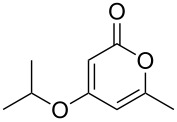 **4a**	70
2	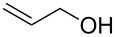 **5b**	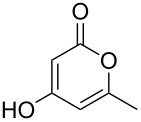 **3a**	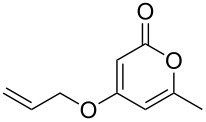 **4b**	54
3	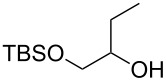 **5c**	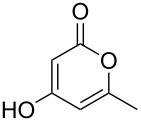 **3a**	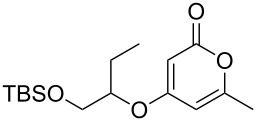 **4c**	98
4	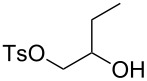 **5d**	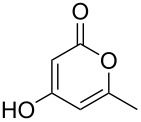 **3a**	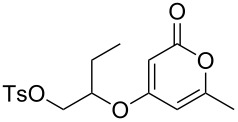 **4d**	99
5	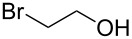 **5e**	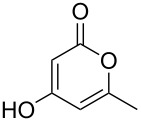 **3a**	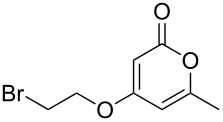 **4e**	82
6	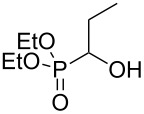 **5f**	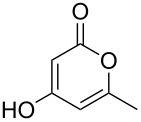 **3a**	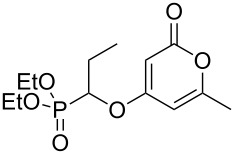 **4f**	30
7	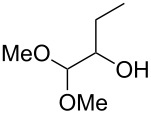 **5g**	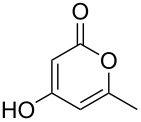 **3a**	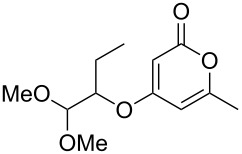 **4g**	23
8	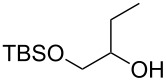 **5b**	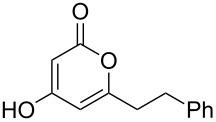 **3b**	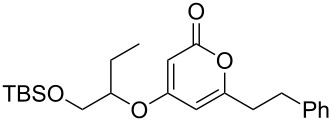 **4h**	81
9	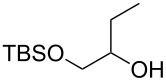 **5b**	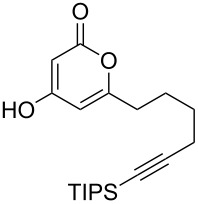 **3c**	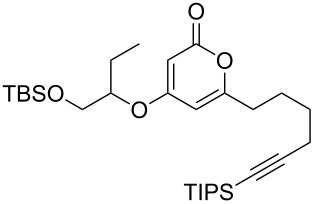 **4i**	75
10	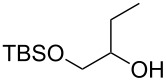 **5b**	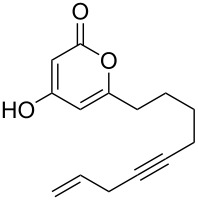 **3d**	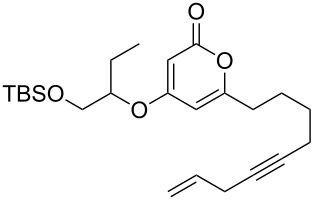 **4j**	50
11	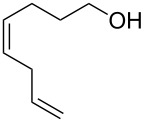 **5h**	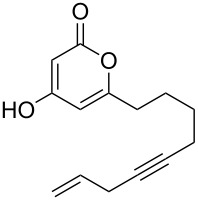 **3d**	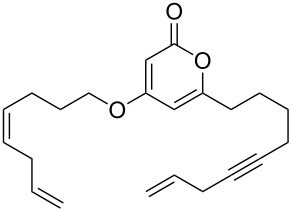 **4k**	52
12	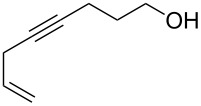 **5i**	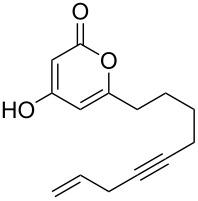 **3d**	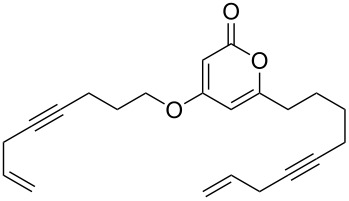 **4l**	61

^a^Yield of product isolated following chromatography on silica gel.

These more structurally complex systems also underwent Mitsunobu reaction with various alcohols in good to excellent yields (Table 1, entries 8–12). Compounds **4i**–**l** could find further utility in the synthesis of phacelocarpus 2-pyrones in the future.

### Oxa-Michael additions

Whilst the formation of pyronyl ethers is useful in itself, the ability to introduce an unsaturated group onto the oxygen, leading to a pyronyl enol ether, would have additional value. This is a highly unusual motif found in some marine polyketide natural products (such as compound **1**, Figure 1). Conjugate addition to α,β-ynones represents an intuitive and efficient route to vinyl compounds, and is well-established with a plethora of oxygen-based nucleophiles [19]. Addition of highly acidic coupling partners can be challenging, however, due to the low nucleophilicity of the conjugate base in which the electron density is extensively delocalised. To the best of our ability we could not find a single previous published example of a Michael addition employing 4-hydroxy-2-pyrones.

Initial experiments reacting 4-hydroxy-2-pyrone **3a** with methyl propiolate (**6a**) in the presence of an amine base afforded only moderate yields of product **7a** (Table 2, entry 1). However, raising the temperature of the reaction and increasing the reaction time led to a significant increase in the conversion to product and an isolated yield of 82% (Table 2, entry 3). Further heating under pressure with microwave irradiation led to a decrease in yield.

**Table 2:** Michael addition reaction optimisation.

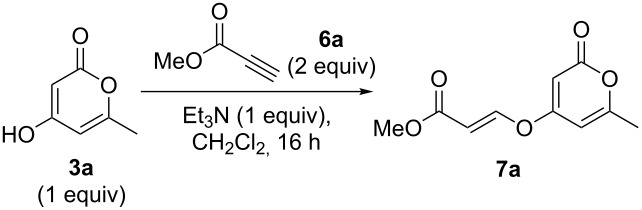			

Entry	Temperature (°C)	Time (h)	Yield (%)^a^

1	20	2	48
2	20	16	63
3	45	16	82
4	80^b^	0.5	67

^a^Yield of product isolated following chromatography on silica gel. ^b^Reaction performed under microwave irradiation.

Following reaction optimisation, we applied these conditions to a number of different propiolate esters and alkylated 4-hydroxy-2-pyrones (Table 3). Surprisingly, the tolerance of the reaction to different ester groups proved rather limited. The switch from methyl propiolate to *tert*-butyl propiolate (**6b**) led to a slight drop in yield (Table 3, entry 2), but moving to pentafluorophenyl propiolate (**6c**) reduced the yield to a modest 27% (Table 3, entry 3). An attempt with *N*-methoxy-*N*-methylpropiolamide (**6d**) led to no product formation (Table 3, entry 4), and significant recovery of starting material. Changes in the C-6 substituent on the pyrone were tolerated much better, with yields from good to excellent for a range of pyrones with methyl propiolate (Table 3, entries 5–8). A further attempt with pentafluorophenyl propiolate resulted in a poor yield (Table 3, entry 9).

**Table 3:** Synthesis of pyronyl vinyl ethers **7**.

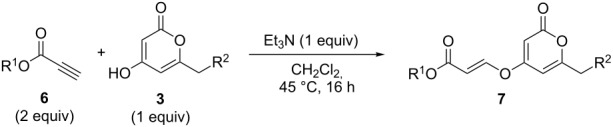				

Entry	Electrophile	Pyrone	Product	Yield (%)^a^

1	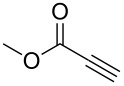 **6a**	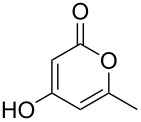 **3a**	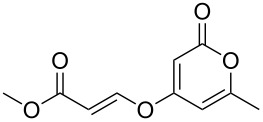 **7a**	82
2	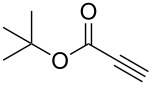 **6b**	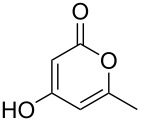 **3a**	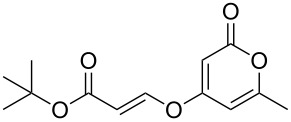 **7b**	61
3	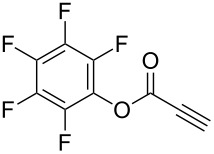 **6c**	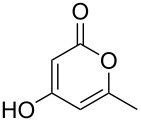 **3a**	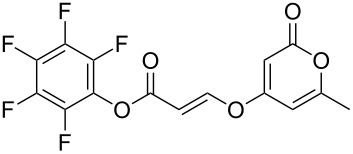 **7c**	27
4	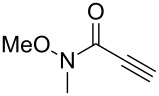 **6d**	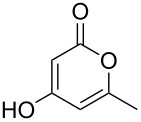 **3a**	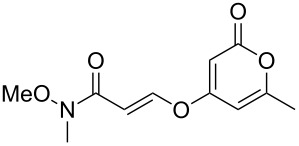 **7d**	0
5	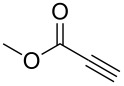 **6a**	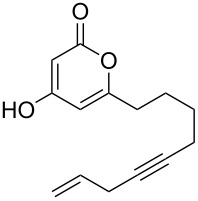 **3d**	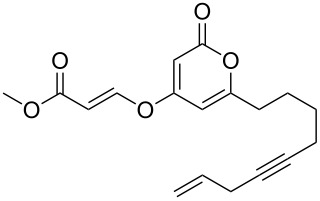 **7e**	64
6	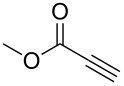 **6a**	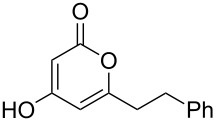 **3b**	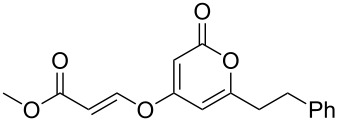 **7f**	68
7	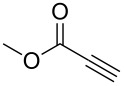 **6a**	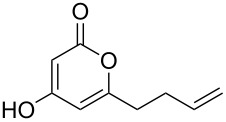 **3e**	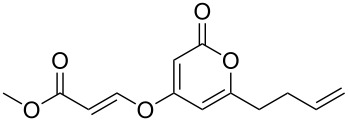 **7g**	86
8	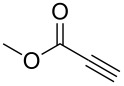 **6a**	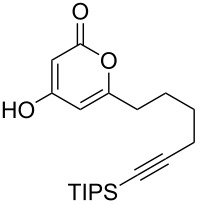 **3c**	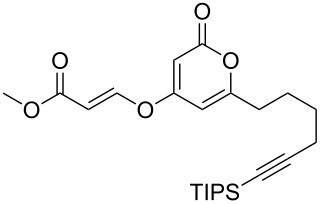 **7h**	82
9	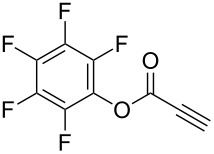 **6c**	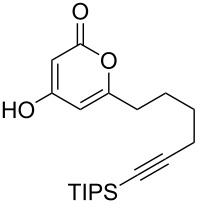 **3c**	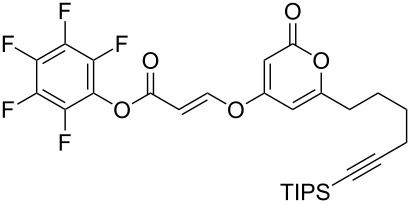 **7i**	37

^a^Yield of product isolated following chromatography on silica gel.

**Scheme 3:**
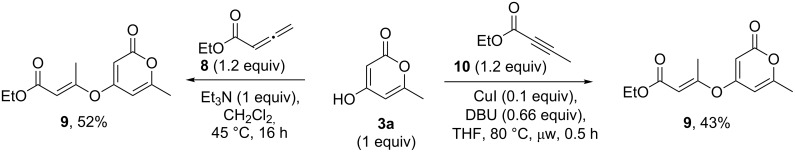
Michael addition of **3a** to allene **8** and internal alkyne **10**.

As an extension to this methodology, we explored the addition of hydroxypyrones to both an allene and an internal alkyne to furnish a trisubstituted enol ether. Addition of 4-hydroxy-6-methyl-2-pyrone (**3a**) to the terminal allene **8** under the optimised conditions proceeded smoothly to give the *trans*-trisubstituted enol ether **9** in 52% yield (Scheme 3). However, numerous attempts to apply these conditions to an internal alkyne failed to furnish any desired product, presumably due to the steric influence of the additional methyl group. However, after an exhaustive screening of conditions (see Supporting Information File 1 for full details), we found that the addition of copper(I) iodide to the reaction mediated the addition of pyrone **3a** to ethyl 2-butynoate (**10**). The reaction was performed with a sub-stoichiometric quantity of DBU (whilst primarily functioning as a base, the DBU was also suspected to be acting partially as a co-solvent as the solubility of the **3a** was found to be greatest at 0.66 equivalents) in THF under microwave irradiation (Scheme 3), and afforded the desired product in moderate yield after just 0.5 h (longer reaction times led to degradation of the product). Extension of the chain to an ethyl group (i.e., using ethyl 2-pentynoate) served to further reduce the reactivity towards addition and no product was formed even under the optimised conditions.

## Conclusion

In conclusion, 4-hydroxy-6-alkyl-2-pyrones are effective coupling partners in Mitsunobu reactions and oxa-Michael additions. The reactions have been shown to tolerate a range of different functional groups by virtue of the mild conditions employed, affording the desired products in moderate to excellent yields in the majority of cases. This protocol offers a practical method for the synthesis of functionalized 2-pyronyl ethers which should find use in the synthesis of natural products and other bioactive compounds.

## Experimental

**General procedure 1: Mitsunobu reaction with 4-hydroxy-2-pyrones:** To a stirred solution of the pyrone (1 equiv), triphenylphosphine (1.5 equiv) and alcohol (1.5 equiv), in dichloromethane (4 mL mmol^−1^) under nitrogen either at 0 °C or ambient temperature, was carefully added DIAD (1.5 equiv) over 10–30 min (depending on scale), so as to avoid the generation of excess heat (<5 °C internal temperature increase). The solution was then stirred at rt (typically 18–25 ºC) for 16 hours, and the solvent removed in vacuo. Byproduct phosphine oxide was removed from the crude residue by dissolving the product in ether (2 mL mmol^−1^), and vacuum filtration to remove the solid oxide. The ether was then removed in vacuo and the residue purified via flash column chromatography to afford the desired product.

**General procedure 2: Oxa-Michael addition with 4-hydroxy-2-pyrones:** The pyrone (1 equiv), triethylamine (1 equiv) and propiolate ester (2 equiv) were stirred in CH_2_Cl_2_ (2 mL mmol^−1^) at 45 °C for 16 h. The solvent was then removed in vacuo and the product purified via flash column chromatography to afford the desired product.

## Supporting Information

File 1 Detailed experimental procedures, characterisation data for compounds **3b–e, 4a–l**, **5d**, **7a–i** and **9** and ^1^H NMR spectra for novel compounds.
