# Supplementary material for: 4-Hydroxy-6-alkyl-2-pyrones as nucleophilic coupling partners in Mitsunobu reactions and oxa-Michael additions
Source: Beilstein J Org Chem. 2014 May 20;10:1159–65. doi: 10.3762/bjoc.10.116 (PMC4077367; doi:10.3762/bjoc.10.116)
Supplement: File 1 — Detailed experimental procedures, characterisation data for compounds 3b–e, 4a–l, 5d, 7a–i and 9 and 1H NMR spectra for novel compounds. [file Beilstein_J_Org_Chem-10-1159-s001.pdf]

Supporting Information

for

4-Hydroxy-6-alkyl-2-pyrones as nucleophilic coupling partners in Mitsunobu reactions and oxa-Michael additions

Michael J. Burns, Thomas O. Ronson, Richard J. K. Taylor and Ian J. S. Fairlamb*

Address: Department of Chemistry, University of York, Heslington, York, YO10 5DD, UK

Email: Ian J. S. Fairlamb* – ian.fairlamb@york.ac.uk

* Corresponding author

Detailed experimental procedures, characterisation data for compounds 3b–e, 4a–l, 5d, 7a–i and 9, and ^{1}H NMR spectra for novel compounds.

1. General Experimental Details……………………………………………………….S2

2. General Procedures……………………………………………………………………..S2

3. Optimisation table for reaction between 3a and 9……………………………....S4

4. Compound Characterisation Data…………………………………………………….S5

5. References……………………………………………………………………………….S18

6. Representative ^{1}H NMR spectra for novel compounds………..……..…S19-S43

1. General Experimental Details

Reagents were purchased from either Sigma Aldrich or Alfa Aesar and used as received. Dry THF was distilled over sodium wire using benzophenone indicator and stored over a potassium mirror. Triethylamine was dried over KOH. Dichloromethane was dried utilising a chromasolv® solvent column. Nitrogen gas was oxygen free and dried immediately before use by passage through sodium hydroxide pellets and silica.

^{1}H NMR and ^{13}C NMR spectra were recorded on a Jeol ECX400 or Jeol ECS400 spectrometer operating at 400 and 100 MHz respectively, or a Bruker 500 spectrometer operating at 500 and 126 MHz respectively. ^{19}F NMR spectra were recorded on a Jeol ECX400 spectrometer at 376 MHz. ^{31}P NMR spectra were recorded on a Jeol ECX400 spectrometer at 162 MHz. Column chromatography was performed using flash silica gel with the solvent systems specified within the text. Mass spectrometry was performed on a Bruker daltronics micrOTOF spectrometer, with <5 ppm error recorded for all HRMS samples. IR was performed on a Jasco FTIR 4100 spectrometer using an ATR attachment. Melting point analyses were performed on a Stuart SMP3 melting point apparatus, using a temperature ramp of 3 °C/minute.

Compounds **5c**[1], **5f**[2], **5g**[3], **5h**[4], **5i**[5] and **6c**[6] were prepared according to literature procedures. Compound **6d** was synthesised from the appropriate acid chloride and methoxylamine hydrochloride with triethylamine according to standard procedure.

2. General Procedures

General Procedure 1: Mitsunobu Reaction with 4-hydroxy-2-pyrones

To a stirred solution of the pyrone (1 eq.), triphenylphosphine (1.5 eq.) and alcohol (1.5 eq.), in dichloromethane (4 mL $mmol^{-1}$) under nitrogen either at 0 °C or ambient temperature, was carefully added DIAD (1.5 eq.) over 10–30 mins (depending on scale), so as to avoid the generation of excess heat (<5 °C internal temperature increase). The solution was then stirred at RT (typically 18–25 ºC) for 16 hours, and the solvent removed in vacuo. By-product phosphine oxide was removed from the crude residue by dissolving the product in ether (2 mL $mmol^{-1}$), and vacuum filtration to remove the solid oxide. The ether was then removed in vacuo and the residue purified via flash column chromatography to afford the desired product.

General Procedure 2: Oxa-Michael Addition with 4-hydroxy-2-pyrones

The 2-pyrone (1 eq.), triethylamine (1 eq.) and propiolate ester (2 eq.) were stirred in CH_2Cl_2 (2 mL $mmol^{-1}$) at 45 °C for 16 hours. The solvent was then removed *in vacuo* and the product purified *via* flash column chromatography to afford the desired product.

General Procedure 3: Lithiation/alkylations of 4-hydroxy-6-methyl-2-pyrone

4-Hydroxy-6-methyl-2-pyrone **3a** (1 mmol, 1 eq.) was heated to 80 °C under nitrogen in HMDS (3 mL) for 1 hour. The solution was allowed to cool and the HMDS removed under vacuum. THF (3 mL) was then added and the solution cooled to −78 °C at which point *n*-BuLi (2.5 M in hexanes, 1.25 mmol, 1.25 eq.) was added carefully over 15 minutes, and the solution stirred for 1 hour. The alkyl halide (1.7–2.3 mmol, 1.7–2.3 eq.) was then added over 10 minutes and the solution allowed to warm gradually to 20 °C and stirred for 16 hours. The reaction was then quenched with 6 M aq. HCl until the pH ≈ 2 and the solvent removed *in vacuo*. The residue was taken up in ethyl acetate (5 mL), washed twice in brine, dried over $MgSO_4$, filtered and concentrated *in vacuo* to afford a crude brown residue which was purified by flash column chromatography (3% MeOH in dichloromethane).

3. Optimisation table for reaction between 3a and 9

Table S1

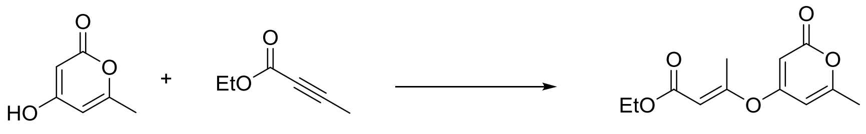

3a **10** **9**

Entry	Solvent	Base (eq.)	Additive (mol%)	Temp. (°C)	Time (h)	Yield[a] (%)
1	CH_2Cl_2	Et_3N (1)	-	40	1	0
2	CH_2Cl_2	Et_3N (1)	-	80 (mw)	1	*ca*. 1
3	CH_2Cl_2	DBU (2)	-	90 (mw)	1	4
4	THF	DBU (1)	BF_3 (20)	90 (mw)	2	18[b]
5	CH_2Cl_2	DBU (1)	BF_3 (20)	80 (mw)	2	6[b]
6	THF	DBU (1)	BF_3 (20)	80 (mw)	2	24[b]
7	THF	Et_3N (1)	BF_3 (20)	80 (mw)	2	5[b]
8	THF	DBU (1)	$Yb(OTf)_3$ (20)	80 (mw)	2	13
9	THF	DBU (1)	BF_3 (20)	70	16	22[b]
10	THF	DBU (1)	BF_3 (6)	80 (mw)	2	19[b]
11	THF	DBU (1)	B(*i*-Pr)$_3$ (20)	80 (mw)	2	17
12	CH_2Cl_2	Et_3N (1)	CuI (10)	80 (mw)	1	29
13	THF	DBU (1)	CuI (10)	40	16	29
14	THF	DBU (1)	CuI (10)	80 (mw)	2	26
15	THF	DBU (0.1)	CuI (10)	80 (mw)	3	0
16	THF	DBU (0.2)	CuI (10)	80 (mw)	6	30
17	THF	DBU (0.66)	CuI (10)	80 (mw)	0.5	43
18	THF	DBU (0.66)	CuI (10)	40	15	7
19	THF	DBU (0.66)	CuI (10)/ B(*i*-Pr)$_3$ (20)	80 (mw)	0.5	9
20	THF	NaH (1)	-	20	5	0
21	THF	NaH (1)	-	40	5	0
22	THF	NaH (1)	-	70	5	0
23	THF	NaH (1)	CuI (10)	70	5	0

[a] Yield of isolated product following column chromatography. [b] Extensive degradation of 3a observed.

4. Compound Characterisation Data

4-Hydroxy-6-(2-phenylethyl)-2-pyrone (3b)[7]

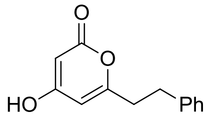

Prepared according to general procedure 3 using **3a** (126 mg, 1 mmol, 1 eq.) and benzyl bromide (373 mg, 2.3 mmol, 2.3 eq.) to afford the title compound as a yellow solid (85 mg, 40%).

M. P. 134–135 °C (lit. 137–138 °C); **^{1}H-NMR** (400 MHz, $CDCl_3$): 7.32–7.14 (m, 5H), 5.92 (d, *J* = 2.1 Hz, 1H), 5.57 (d, *J* = 2.1 Hz, 1H), 2.96 (dd, *J* = 8.8, 6.8 Hz, 2H), 2.78 (dd, *J* = 8.8, 6.8 Hz, 2H); **^{13}C-NMR** (100 MHz, $CDCl_3$): 172.24, 167.94, 165.87, 139.50, 128.54, 128.15, 126.43, 101.72,89.94, 35.36, 32.70; **MS** (ESI) *m/z* (rel. %): 217 [MH^+] (100); **HRMS** (ESI) calculated for $C_{13}H_{13}O_3$ [MH^+]: 217.0859, found: 217.0865.

4-Hydroxy-6-(6-triisopropylsilyl-hex-5-yne)-2-pyrone (3c)

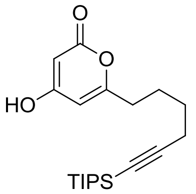

Prepared according to general procedure 3 using **3a** (126 mg, 1 mmol, 1 eq.) and 5-triisopropylsilyl-1-iodopent-4-yne[8] (550 mg, 1.6 mmol, 1.6 eq.) to afford the title compound as a yellow oil (239 mg, 69%).

^{1}H-NMR (400 MHz, $CDCl_3$): 5.93 (d, *J* = 2.1 Hz, 1H), 5.54 (d, *J* = 2.1 Hz, 1H), 2.47 (t, *J* = 7.5 Hz, 2H), 2.24 (t, *J* = 6.9 Hz, 2H), 1.74–1.84 (m, 2H), 1.53–1.62 (m, 2H), 0.97–1.01 (m, 21H); **^{13}C-NMR** (100 MHz, $CDCl_3$): 172.3, 167.9, 166.8, 107.9, 101.3, 89.9, 80.9, 53.4, 33.0, 27.9, 25.6, 18.6, 11.2; **MS** (ESI) *m/z* (rel. %): 349 [MH^+] (100), 307 (8), 255 (3), 167 (4); **HRMS** (ESI) calculated for $C_{20}H_{33}O_3Si$ [MH^+]: 349.2193, found: 349.2189; **IR** (CH_2Cl_2, cm^{-1}): 3684, 2944, 2865, 2169, 1693, 1613, 1572, 1462.

1-Iodooct-7-en-4-yne (11)

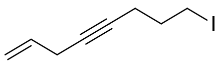

To a solution of oct-7-en-5-yn-1-ol (**5i**)[5] (12.4 g, 100 mmol, 1 eq.) and triethylamine (20.8 g, 150 mmol, 1.5 eq.) in dichloromethane (800 mL) under nitrogen at 20 °C, was added

methanesulfonylchloride (22.9 g, 200 mmol, 2 eq.). The reaction was quenched with ice cold water (150 mL) and separated. The organic layer was then washed with cold 2 M HCl (150 mL), sat. $NaHCO_3$ (150 mL) and brine (150 mL). The organic extracts were dried over $MgSO_4$, filtered and concentrated *in vacuo* to afford the crude mesylate as a pale yellow oil (20.2 g, >99%) which was used without further purification.

A solution of the crude mesylate (20.2 g, 100 mmol, 1 eq.) and NaI (45 g, 300 mmol, 3 eq.) was stirred in acetone (300 mL) for 72 hours under nitrogen, and then heated to reflux for 2 hours. The solution was allowed to cool, filtered, and the filtrate concentrated *in vacuo*. The crude product was then taken up in hexane (3 × 40 mL), filtered, and reduced *in vacuo* to afford a pale yellow oil (17.4 g, 74.5%), which was used immediately without further purification.

^{1}H-NMR (400 MHz, $CDCl_3$): 5.81 (ddt, *J* = 16.9, 10.0, 5.3 Hz, 1H), 5.30 (ddt, *J* =16.9, 1.7, 1.7 Hz, 1H), 5.10 (ddt, *J* = 10.0, 1.7, 1.7 Hz, 1H), 3.31 (t, *J* = 6.7 Hz, 2H), 2.93 (m, 2H), 2.34 (tt, *J* = 6.7, 2.4 Hz, 2H), 1.99 (p, *J* = 6.7 Hz, 2H); **^{13}C-NMR** (100 MHz, $CDCl_3$): 133.50, 116.15, 80.77, 78.35, 32.86, 23.49, 20.25, 5.90; **MS** (EI) *m/z* (rel.%): 234 [MH^+] (33), 206 (21), 155 (10), 127 (8), 105 (9), 91 (45), 79 (100), 65 (10), 51 (25), 39 (23); **IR** (neat, cm^{-1}): 2945, 2865, 2173, 1463, 1427, 1364, 1220, 1177.

4-Hydroxy-6-(non-8-en-5-ynyl)-2-pyrone (3d)

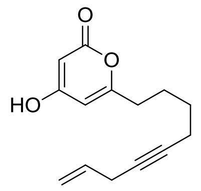

Prepared according to general procedure 3 using **3a** (126 mg, 1 mmol, 1 eq.) and 1-iodooct-7-en-4-yne **11** (330 mg, 1.4 mmol, 1.4 eq.) to afford the title compound as a yellow oil (74 mg, 32%).

^{1}H-NMR (400 MHz, $CDCl_3$): 6.00 (d, *J* = 2.1 Hz, 1H), 5.83 (ddt, *J* = 17.0, 10.0, 5.3 Hz, 1H), 5.58 (d, *J* = 2.1 Hz, 1H), 5.28 (ddt, *J* = 17.0, 1.8, 1.8 Hz, 1H), 5.08 (ddt, *J* = 10.0, 1.8, 1.8 Hz, 1H), 2.91–2.94 (m, 2H), 2.51 (t, *J* = 7.6, 2H), 2.22 (tt, *J* = 7.0, 2.4 Hz, 2H), 1.72–1.80 (m, 2H) 1.51–1.58 (m, 2H); **^{13}C-NMR** (100 MHz, $CDCl_3$) 172.6, 168.2, 166.9, 133.2, 133.2, 115.7, 115.7, 101.5, 89.9, 81.8, 33.2, 28.1, 25.7, 23.1, 18.4; **MS** (ESI) *m/z* (rel. %): 233 [MH^+] (100), 255 [MNa^+] (19); **HRMS** (ESI) calculated for $C_{14}H_{17}O_3$ [M^+]: 233.1172, found: 233.1178; **IR** (neat, cm^{-1}): 3083, 2939, 2619, 1694, 1568, 1492, 1445, 1364, 1250, 1142, 993.

4-Hydroxy-6-(but-3-en)-2-pyrone (3e)[7]

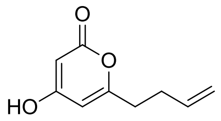

Prepared according to general procedure 2 using **3a** (126 mg, 1 mmol, 1 eq.) and allyl bromide (363 mg, 2.3 mmol, 2.3 eq.) to afford the title compound as a waxy yellow solid (88 mg, 50%).

^{1}H-NMR (400 MHz, $CDCl_3$): 6.01 (d, *J* = 1.9 Hz, 1H), 5.77 (ddt, *J* = 17.1, 10.1, 6.8 Hz, 1H), 5.58 (d, *J* = 1.9 Hz, 1H), 5.05 (ddt, *J* = 17.1, 1.5, 1.5 Hz, 1H), 5.01 (dtd, *J* = 10.1, 3.3, 1.5 Hz, 1H), 2.57 (t, *J* = 7.5 Hz, 2H), 2.35–2.41 (m, 2H); **^{13}C-NMR** (100 MHz, $CDCl_3$): 172.70, 168.38, 166.28, 135.94, 116.45, 101.85, 90.02, 33.04, 30.62; **MS** (ESI) *m/z* (rel.%): 167 [MH^+] (100); **HRMS** (ESI) calculated for $C_9H_{11}O_3$ [MH^+]: 167.0703, found: 167.0704.

4-Isopropyloxy-6-methyl-2-pyrone (4a)

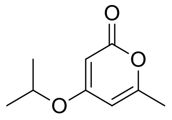

Synthesised using general procedure 1 and purified by flash column chromatography (20% EtOAc in hexanes) to afford a white crystalline solid (1.76 g, 70%)

M. P. 49–51 °C; **^{1}H-NMR** (400 MHz, $CDCl_3$): 5.71 (m, 1H), 5.35 (d, *J* = 2.2 Hz, 1H), 4.49 (sept., *J* = 6.1 Hz, 1H), 2.18 (s, 3H), 1.32 (d, *J* = 6.1 Hz, 6H); **^{13}C-NMR** (100 MHz, $CDCl_3$): 169.4, 165.2, 161.9, 101.0, 87.8, 71.4, 21.2, 19.7; **MS** (ESI) *m/z* (rel.%): 169 [MH^+] (100). **HRMS** (ESI) calculated for $C_9H_{12}O_3Na$ [M^+]: 191.0679, found: 191.0682; **IR** (CH_2Cl_2, cm^{-1}): 2984, 2937, 1733, 1651, 1562, 1467, 1376, 1321.

4-Prop-2-enoxy-6-methyl-2-pyone (4b)[9]

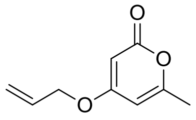

Synthesised using general procedure 1 and purified by flash column chromatography (20% EtOAc in hexane) to afford the title compound as a colourless oil (0.90 g, 54%).

^{1}H-NMR (400 MHz, $CDCl_3$): 5.94 (ddt, *J* = 17.3, 10.8, 5.5 Hz, 1H), 5.78 (dd, *J* = 2.2, 1.0 Hz, 1H), 5.38 (dq, *J* = 17.3, 1.5 Hz, 1H), 5.37 (d, *J* = 2.2 Hz, 1H), 5.32 (dq, *J* = 10.8, 1.3 Hz, 1H), 4.47 (dt, *J* = 5.5, 1.3 Hz, 2H), 2.18 (s, 3H); **^{13}C-NMR** (100 MHz, $CDCl_3$): 170.2, 165.0, 162.3,

130.9, 119.4, 100.6, 88.3, 69.4, 19.9; **MS** (ESI) *m/z* (rel. %): 189 [MNa$^+$] (100), 167 [MH$^+$] (45); **HRMS** (ESI) calculated for $C_9H_{10}O_3Na$: 189.0522, found: 189.0525; **IR** (thin film, cm^{-1}): 1708, 1649, 1561, 1449, 1410, 1319, 1246, 1182, 1142, 942, 859, 810, 684, 543.

4-[1-(*tert*-Butyl-dimethyl-silanyloxymethyl)-propoxy]-6-methyl-2-pyrone (4c)[1]

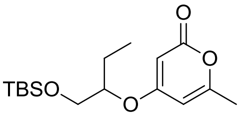

Synthesised using general procedure 1 and purified *via* flash column chromatography (20% EtOAc in hexanes) to afford a colourless oil (1.23 g, 98.4%).

R$_f$ = 0.56 (20% EtOAc); **^{1}H-NMR** (400 MHz, $CDCl_3$): 5.71 (dq, *J*= 1.9, 0.8 Hz, 1H), 5.36 (d, *J* = 1.9 Hz, 1H), 4.17 (p, *J* = 5.4 Hz, 1H), 3.66 (s, 1H), 3.64 (d, *J* = 1.3 Hz, 1H), 2.11–2.15 (m, 3H), 1.53–1.69 (m, 2H), 0.87 (t, *J* = 7.5 Hz, 3H), 0.79 (s, 9H), −0.03 (s, 3H), −0.04 (s, 3H); **^{13}C-NMR** (100 MHz, $CDCl_3$): 170.5, 165.3, 162.1, 100.9, 88.4, 80.9, 63.8, 25.8, 23.4, 19.8, 18.2, 9.5, −5.4; **MS** (ESI) *m/z* (rel.%): 335 [MNa$^+$] (100), 313 [MH$^+$] (2); **HRMS** (ESI) calculated for $C_{16}H_{28}O_4NaSi$ [M$^+$]: 335.1649, found: 335.1640; **IR** (neat, cm^{-1}): 2929, 2883, 2857, 1736, 1651, 1563, 1450, 1414, 1320, 1249, 1139, 1036, 1001.

4-(1-Toluenesulfonyloxybutyl-2-oxy)-6-methyl-2-pyrone (4d)

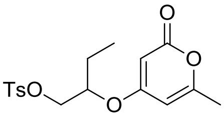

Synthesised using general procedure 1 and purified by flash column chromatography (10–40% EtOAc in heptane), to afford the title compound as a white crystalline solid (38.4 g, 99%).

M. P. 96–98 °C; **^{1}H-NMR** (400 MHz, $CDCl_3$): 7.73 (d, *J* = 7.5 Hz, 2H), 7.31 (d, *J* = 7.5 Hz, 2H), 5.67–5.64 (m, 1H), 5.22 (d, *J* = 2.1 Hz, 1H) 4.33–4.28 (m, 1H), 4.16–4.07 (m, 2H), 2.42 (s, 3H), 2.15 (s, 3H), 1.68 (p, *J* = 7.5 Hz, 2H), 0.93 (t, *J* = 7.5 Hz, 3H); **^{13}C-NMR** (100 MHz, $CDCl_3$): 169.2, 164.6, 162.4, 145.3, 130.0, 127.9, 100.5, 88.4, 68.8, 60.4, 23.1, 21.7, 19.8, 14.2, 9.0; **MS** (ESI) *m/z* (rel. %): 375 [MNa$^+$] (100), 353 [MH$^+$] (80), 281 (7), 227 (20); **HRMS** (ESI) calculated for $C_{17}H_{21}O_6S$ [MH$^+$]: 353.1053, found: 353.1062; **IR** (CH_2Cl_2, cm^{-1}): 3062, 2979, 2883, 1713, 1652, 1598, 1436, 1365, 1243, 1178, 1096.

4-(2-Bromoethoxy)-6-methyl-2-pyrone (4e)

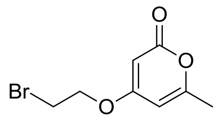

Synthesised using general procedure 1 and purified by flash column chromatography (10–40% EtOAc in heptane) to afford the title compound as a pale yellow crystalline solid (19.1 g, 82%).

M. P. 66–68 °C; **^{1}H-NMR** (400 MHz, $CDCl_3$): 5.83 (m, 1H), 5.37 (t, *J* = 2.5 Hz, 1H), 4.26 (t, *J* = 6.0 Hz, 2H), 3.61 (t, *J* = 6.0 Hz, 2H), 2.21 (s, 3H); **^{13}C-NMR** (100 MHz, $CDCl_3$): 169.7, 164.5, 162.5, 100.1, 88.1, 68.0, 27.1, 19.8; **MS** (ESI) *m/z* (rel. %): 235 [MH^+(^{81}Br)] (84), 233 [MH^+(^{79}Br)] (100); **HRMS** (ESI) calculated for $C_8H_9{}^{79}BrO_3Na$: 254.9627, found: 254.9622; **IR** (CH_2Cl_2, cm^{-1}): 3059, 1716, 1653, 1571, 1448, 1418, 1377, 1251, 1184, 1145.

4-[1-(Diethylphosphono)-propoxy]-6-methyl-2-pyrone (4f)

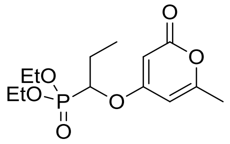

Synthesised using general procedure 1 and purified by flash column chromatography (EtOAc in hexanes) to afford the title compound as a colourless oil (91 mg, 30%).

^{1}H-NMR (400 MHz, $CDCl_3$): 5.84 (dd, *J* = 2.2, 1.0 Hz, 1H), 5.47 (d, *J* = 2.2 Hz, 1H), 4.37 (td, *J* = 8.2, 4.6 Hz, 1H), 4.21–4.08 (m, 4H), 2.20 (s, 3H), 2.07–1.84 (m, 2H), 1.32 (t, *J* = 7.0 Hz, 3H), 1.30 (t, *J* = 7.0 Hz, 3H), 1.01 (dt, *J* = 0.7, 7.4 Hz, 3 H); **^{13}C-NMR** (101 MHz, $CDCl_3$): 170.1, 164.8, 162.7, 100.5, 89.1, 75.5 (d, $^{1}J_{C\text{–}P}$ = 169.3 Hz), 63.3 (d, $^{2}J_{C\text{–}P}$ = 6.9 Hz), 62.9 (d, $^{2}J_{C\text{–}P}$ = 7.2 Hz), 23.3, 20.0, 16.6 (app. t, $^{3}J_{C\text{–}P}$ = 5.4 Hz), 10.3 (d, $^{2}J_{C\text{–}P}$ = 11.3); **^{31}P-NMR** (162 MHz, $CDCl_3$): 19.7 (s); **MS** (ESI): *m/z* (rel. %) 327 $[MNa]^+$ (100), 305 $[MH]^+$ (25); **HRMS** *m/z* $[MNa]^+$ calcd for $C_{13}H_{21}NaO_6P$: 327.0968; found: 327.0970; **IR** (neat, cm^{-1}) 1712, 1648, 1564, 1448, 1408, 1242, 1143, 1016, 963, 810, 559, 542.

4-[1-(Dimethoxymethyl)-propoxy]-6-methyl-2-pyrone (4g)

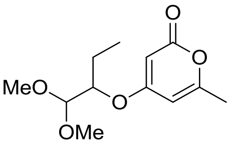

Synthesised using general procedure 1 and purified by flash column chromatography (20% EtOAc in hexanes) to afford the title compound as a colourless oil (117 mg, 23%). Due to the limited stability of the compound, only partial data could be obtained.

^{1}H-NMR (500 MHz, $CDCl_3$): 5.81 (dq, *J* = 2.3, 0.9 Hz, 1H), 5.46 (d, *J* = 2.3 Hz, 1H), 4.34 (d, *J* = 5.3 Hz, 1H), 4.20 (ddd, *J* = 7.5, 5.3, 4.3 Hz, 1H), 3.42 (s, 3H), 3.40 (s, 3H), 2.19 (d, *J* = 0.9 Hz, 3H), 1.62–1.86 (m, 2H), 0.93 (t, *J* = 7.5 Hz, 3H); **^{13}C-NMR** (126 MHz, $CDCl_3$): 170.3, 165.2, 162.2, 104.4, 100.7, 88.6, 79.5, 55.6, 55.5, 22.4, 19.8, 9.3.

4-[1-(*tert*-Butyl-dimethyl-silanyloxymethyl)-propoxy]-6-(2-phenylethyl)-2-pyrone (4h)

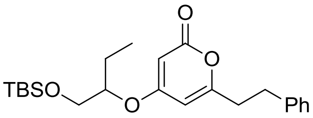

Synthesised using general procedure 1 and purified by flash column chromatography (20% EtOAc in hexanes) to afford the title compound as a colourless oil (45 mg, 81%).

^{1}H-NMR (500 MHz, $CDCl_3$): 7.26 (t, *J* = 7.4 Hz, 2H), 7.21 (t, *J* = 7.4 Hz, 1H), 7.17 (d, *J* = 7.4 Hz, 2H), 5.71 (d, *J* = 2.1 Hz, 1H), 5.44 (d, *J* = 2.1 Hz, 1H), 4.22 (p, *J* = 5.5 Hz, 1H), 3.67–3.73 (m, 2H), 2.97 (t, *J* = 7.9 Hz, 2H), 2.74 (t, *J* = 7.9 Hz, 2H), 1.59–1.76 (m, 2H), 0.93 (t, *J* = 7.5 Hz, 3H), 0.86 (s, 9H), 0.03 (s, 3H), 0.01 (s, 3H); **^{13}C-NMR** (126 MHz, $CDCl_3$): 170.3, 165.2, 164.4, 139.9, 128.6, 128.3, 126.4, 100.8, 88.8, 80.9, 63.8, 35.5, 32.9, 25.8, 23.4, 18.2, 9.4, −5.4, −5.5; **MS** (ESI): *m/z* (rel. %) 425 $[MNa]^+$ (19), 403 $[MH]^+$ (100), 391 (6); **HRMS** *m/z* $[MH]^+$ calcd for $C_{23}H_{35}O_4Si$: 403.2299; found: 403.2300; **IR** (neat, cm^{-1}) 2956, 2929, 2857, 1718, 1649, 1564, 1471, 1423, 1249, 1132, 1107, 838, 755.

4-[1-(*tert*-Butyl-dimethyl-silanyloxymethyl)-propoxy]-6-(6-triisopropylsilyl-hex-5-ynyl)-2-pyrone (4i)

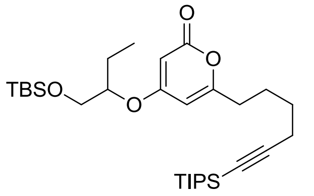

Synthesised using general procedure 1 and purified *via* flash column chromatography (20% EtOAc in hexanes) to afford a colourless oil (628 mg, 75%).

^{1}H-NMR (500 MHz, $CDCl_3$): 5.77 (d, *J* = 2.3 Hz, 1H), 5.44 (d, *J* = 2.3 Hz, 1H), 4.23 (p, *J* = 5.5 Hz, 1H), 3.68–3.74 (m, 2H), 2.47 (t, *J* = 7.5 Hz, 2H), 2.29 (t, *J* = 6.9 Hz, 2H), 1.76–1.83 (m, 2H), 1.62–1.75 (m, 2H), 1.54–1.61 (m, 2H), 0.97–1.08 (m, 21H), 0.94 (t, *J* = 7.5 Hz, 3H), 0.86 (s, 9H), 0.03 (s, 3H), 0.02 (s, 3H); **^{13}C-NMR** (126 MHz, $CDCl_3$): 170.4, 165.4, 165.4, 108.4, 100.3, 88.5, 80.8, 77.2, 63.7, 32.9, 27.9, 25.7, 25.6, 23.3, 19.5, 18.6, 18.2, 11.2, 9.4, −5.4, −5.5; **MS** (ESI) *m/z* (rel. %): 557 [MNa^+] (19), 535 [MH^+] (100), 413 (11), 391 (39); **HRMS** (ESI) calculated for $C_{30}H_{55}O_4Si_2$ [MH^+]: 535.3633, found: 535.3648.

4-[1-(*tert*-Butyl-dimethyl-silanyloxymethyl)-propoxy]-6-(non-8-en-5-ynyl)-2-pyrone (4j)

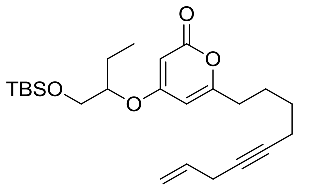

Synthesised using general procedure 1 and purified *via* flash column chromatography (20% EtOAc in hexanes) to afford a colourless oil (93 mg, 49.5%).

^{1}H-NMR (500 MHz, $CDCl_3$): 5.81 (ddt, *J* = 16.9, 10.0, 5.3 Hz, 1H), 5.76 (d, *J* = 2.2 Hz, 1H), 5.42 (d, *J* = 2.2 Hz, 1H), 5.28 (ddt, *J* = 16.9, 1.8, 1.8 Hz, 1H), 5.08 (ddt, *J* = 10.0, 1.8, 1.8 Hz, 1H), 4.22 (p, *J* = 5.5 Hz, 1H), 3.69–3.72 (m, 2H), 2.90–2.94 (m, 2H), 2.45 (t, *J* = 5.5 Hz, 2H), 2.22 (tt, *J* = 7.0, 2.4 Hz, 2H), 1.72–1.80 (m, 2H), 1.62–1.71 (m, 2H), 1.51–1.58 (m, 2H), 0.94 (t, *J* = 7.5 Hz, 3H), 0.86 (s, 9H), 0.03 (s, 3H), 0.02 (s, 3H); **^{13}C-NMR** (126 MHz, $CDCl_3$): 170.3, 165.3, 165.2, 133.2, 115.6, 100.3, 88.6, 81.8, 80.8, 63.7, 33.1, 28.1, 25.7, 23.4, 23.1, 21.9, 20.4, 18.5, 18.2, 9.4, −5.4, −5.5; **MS** (ESI) *m/z* (rel. %): 441 [MNa^+] (60), 419 [MH^+] (100), 391 (9); **HRMS** (ESI) calculated for $C_{24}H_{39}O_4Si$ [MH^+]: 419.2612, found: 419.2620; **IR** (neat, cm^{-1}): 2930, 2857, 1733, 1652, 1559, 1472, 1419, 1241, 1107, 1005, 837.

6-(Non-8-en-5-ynyl)-4-(octa-*Z*-4,7-dienyloxy)-2-pyrone (4k)

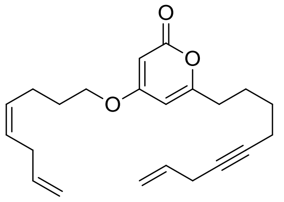

Synthesised using general procedure 1 and purified *via* flash column chromatography (20% EtOAc in hexanes) to afford a colourless oil (25 mg, 52%).

^{1}H-NMR (500 MHz, $CDCl_3$): 5.94 (d, *J* = 2.1 Hz, 1H), 5.74–5.86 (m, 2H), 5.52–5.41 (m, 2H), 5.40 (d, *J* = 2.1 Hz, 1H), 5.29 (ddt, *J* = 17.0, 1.7, 1.7 Hz, 1H), 5.09 (ddt, *J* = 10.0, 1.7, 1.7 Hz, 1H), 5.02 (ddt, *J* = 17.1, 3.4, 1.7 Hz, 1H), 4.98 (ddt, *J* = 10.2, 3.4, 1.6 Hz, 1H), 3.94 (t, *J* = 6.4 Hz, 2H), 2.91–2.96 (m, 2H), 2.74–2.83 (m, 2H), 2.15–2.27 (m, 4H), 1.89–2.06 (m, 2H), 1.80–1.88 (m, 2H), 1.56–1.71 (m, 4H); **^{13}C-NMR** (126 MHz, $CDCl_3$): 170.2, 166.1, 164.2, 136.6, 128.9, 128.8, 128.5, 115.7, 114.9, 100.1, 88.7, 81.6, 77.5, 68.2, 48.0, 33.1, 31.4, 31.1, 28.2, 23.2, 23.1, 14.4; **MS** (ESI) *m/z* (rel. %): 363 [MNa^+] (23), 341 [MH^+] (36), 329 (69), 307 (100); **HRMS** (ESI) calculated for $C_{22}H_{29}O_3$: 341.2111, found: 341.2114.

6-(Non-8-en-5-ynyl)-4-(oct-7-en-4-ynyloxy)-2-pyrone (4l)

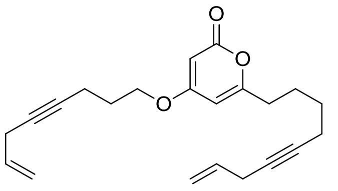

Synthesised using general procedure 1 and purified *via* flash column chromatography (20% EtOAc in hexanes) to afford a colourless oil (59.3 mg, 61%).

^{1}H-NMR (500 MHz, $CDCl_3$): 5.76–5.87 (m, 3H), 5.41 (d, *J* = 2.2 Hz, 1H), 5.29 (ddt, *J* = 16.9, 1.8, 1.8 Hz, 1H), 5.28 (ddt, *J* = 16.9, 1.8, 1.8 Hz, 1H), 5.09 (app. dp, *J* = 10.0, 1.8 Hz, 2H), 4.05 (t, *J* = 6.2 Hz, 2H), 2.91–2.95 (m, 4H), 2.46 (t, *J* = 7.5 Hz, 2H), 2.37 (tt, *J* = 6.8, 2.4 Hz, 2H), 2.23 (tt, *J* = 7.0, 2.4 Hz, 2H), 1.96 (p, *J* = 6.6 Hz, 2H), 1.72–1.80 (m, 2H), 1.51–1.59 (m, 2H); **^{13}C-NMR** (126 MHz, $CDCl_3$): 170.5, 165.3, 165.1, 133.2, 133.0, 115.8, 115.77, 100.0, 88.1, 81.9, 80.5, 78.0, 67.2, 62.0, 33.1, 28.1, 27.8, 25.7, 23.1, 23.0, 18.5, 15.3; **MS** (ESI) *m/z* (rel.%): 339 [MH^+] (48), 333 (69), 313 (100); **HRMS** (ESI) calculated for $C_{22}H_{27}O_3$: 339.1955, found: 339.1949; **IR** (neat, cm^{-1}): 2939, 1700, 1642, 1564, 1436, 1249, 914, 733.

1-Toluenesulfonyloxybutan-2-ol (5d)[10]

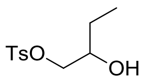

A solution of toluenesulfonyl chloride (51.5 g, 270 mmol, 1 eq.), triethylamine (54.6 g, 405 mmol, 1.5 eq.) and butane-1,2-diol (36.5 g, 540 mmol, 2 eq.) under nitrogen in dichloromethane (800 mL), was stirred at 20 °C for 16 h. The reaction was then quenched with ice cold water (550 mL) and the aqueous layer removed. The organic layer was then washed sequentially with ice-cold 2 M HCl (550 mL), sat. $NaHCO_3$ (550 mL) and brine (550 mL). The organic phase was then dried over $MgSO_4$, filtered and concentrated *in vacuo* to afford the crude material. The product was purified *via* flash column chromatography (10% EtOAc in toluene) to afford a white crystalline powder (53 g, 80%).

M. P. 59–61 °C (lit. 59–60 °C); **^{1}H-NMR** (400 MHz, $CDCl_3$): 7.80 (d, *J* = 8.1 Hz, 2H), 7.35 (d, *J* = 8.1 Hz, 2H), 4.05 (dd, *J* = 10.1, 3.1 Hz, 1H), 3.90 (dd, *J* = 7.1, 10.1 Hz, 1H), 3.77 (qd, *J* = 7.1, 3.1 Hz, 1H), 2.45 (s, 3H), 1.51–1.44 (m, 2H), 0.93 (t, *J = 7.5* Hz, 3H); **^{13}C-NMR** (100 MHz, $CDCl_3$): 145.02, 132.78, 129.92, 127.94, 73.62, 70.79, 25.75, 21.62, 9.56; **MS** (ESI) *m/z* (rel. %): 245 [MH^+] (72), 227 (13), 173 (21); **IR** (neat, cm^{-1}): 3536, 2968, 2881, 1598, 1456, 1359, 1161, 1097, 963.

Methyl 3-(6-methyl-2-pyronyl-4-oxy)-acrylate (7a)

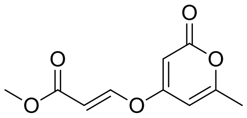

Synthesised using general procedure 2 and purified by column chromatography (20% EtOAc in hexanes) to afford a yellow crystalline powder (12.85 g, 82%).

M. P. 129–130 °C; **^{1}H-NMR** (400 MHz, $CDCl_3$): 7.67 (d, *J* = 12.0 Hz, 1H), 5.91 (dd, *J* = 2.2, 1.0 Hz, 1H), 5.85 (d, *J* = 12.0 Hz, 1H), 5.65 (d, *J* = 2.2 Hz, 1H), 3.78 (s, 3H), 2.28–2.26 (m, 3H); **^{13}C-NMR** (100 MHz, $CDCl_3$); 167.2, 165.9, 164.2, 163.3, 152.5, 107.7, 99.1, 92.1, 48.8, 20.1; **MS** (ESI) *m/z* (rel. %): 233 [MNa^+] (100), 177 (5); **HRMS** (ESI) calculated for $C_{10}H_{10}O_5Na$ [M^+]: 233.0420, found: 233.0429; **IR** (CH_2Cl_2, cm^{-1}): 3061, 2953, 1723, 1644, 1576, 1447, 1406, 1258, 1186, 1101.

tert-Butyl 3-(6-methyl-2-pyronyl-4-oxy)-acrylate (7b)

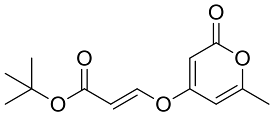

Synthesised using general procedure 2 and purified *via* flash column chromatography (20% EtOAc in hexanes) to afford a yellow crystalline powder (98 mg, 61%).

M. P. 60–62 °C; **^{1}H-NMR** (400 MHz, $CDCl_3$): 7.54 (d, *J* = 12.0 Hz, 1H), 5.90 (d, *J* = 2.2 Hz, 1H), 5.74 (d, *J* = 12.0 Hz, 1H), 5.63 (d, *J* = 2.2 Hz, 1H), 2.25 (s, 3H), 1.48 (s, 9H); **^{13}C-NMR** (101 MHz, $CDCl_3$): 167.3, 164.7, 164.0, 163.6, 151.5, 110.0, 99.3, 91.8, 81.3, 28.1, 20.1; **MS** (ESI) *m/z* (rel. %): 275 [MNa^+] (34), 253 [MH^+] (100), 197 (8); **HRMS** (ESI) calculated for $C_{13}H_{17}O_5$: 253.1071, found: 253.1068; **IR** (neat, cm^{-1}): 3082, 2980, 2160, 1720, 1696, 1658, 1623, 1561, 1235, 1149, 1091, 845.

Pentafluorophenyl 3-(6-methyl-2-pyronyl-4-oxy)-acrylate (7c)

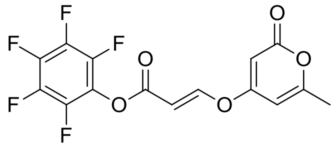

Synthesised using general procedure 2 and purified *via* flash column chromatography (20% EtOAc in hexanes) to afford a yellow crystalline powder (102 mg, 27%).

M. P. 102–105 °C; **^{1}H-NMR** (400 MHz, $CDCl_3$): 7.93 (d, *J* = 12.0 Hz, 1H), 6.05 (d, *J* = 12.0 Hz, 1H), 5.96 (dq, *J* = 2.2, 0.9 Hz, 1H), 5.74 (d, *J* = 2.2 Hz, 1H), 2.29 (t, *J* = 0.9 Hz, 3H); **^{13}C-NMR** (101 MHz, $CDCl_3$): 166.7, 164.7, 163.1, 161.5, 155.9, 104.3, 98.9, 92.9, 20.2 (carbons on perfluorophenyl ring not observed); **^{19}F-NMR** (376 MHz, $CDCl_3$): −152.30–−152.41 (m, 2F), −157.31 (t, *J* = 21.7, 1F), −161.82–−161.99 (m, 2F); **MS** (ESI) *m/z* (rel. %): 380 [MNa^+] (52), 363 [MH^+] (100), 301 (14), 279 (26); **HRMS** (ESI) calculated for $C_{15}H_8F_5O_5$ [MH^+]: 363.0286, found: 363.0299; **IR** (neat, cm^{-1}): 3106, 2916, 2160, 1731, 1641, 1577, 1516, 1280, 1227, 1185, 997.

Methyl 3-(6-(non-8-en-5-ynyl)-2-pyronyl-4-oxy)-acrylate (7e)

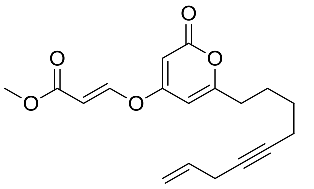

Synthesised using general procedure 2 and purified *via* flash column chromatography (20% EtOAc in hexanes) to afford a waxy yellow powder (28.9 mg, 64%).

^{1}H-NMR (400 MHz, $CDCl_3$): 7.68 (d, *J* = 12.0 Hz, 1H), 5.92 (dt, *J* = 2.3, 0.7 Hz, 1H), 5.85 (d, *J* = 12.0 Hz, 1H), 5.82 (ddt, *J* =16.9, 10.0, 5.3 Hz, 1H), 5.66 (d, *J* = 2.3 Hz, 1H), 5.29 (ddt, *J* = 16.9, 1.8, 1.8 Hz, 1H), 5.09 (ddt, *J* = 10.0, 1.8, 1.8 Hz, 1H), 3.77 (s, 3H), 2.91–2.96 (m, 2H), 2.53 (dt, *J* = 7.6, 0.7 Hz, 2H), 2.24 (tt, *J* = 2.4, 6.9 Hz, 2H), 1.74–1.83 (m, 2H), 1.51–1.62 (m, 2H, C^9H_2); **^{13}C-NMR** (101 MHz, $CDCl_3$): 167.5, 167.1, 166.0, 163.5, 152.4, 133.2, 115.7, 107.7, 99.9, 98.6, 92.1, 81.7, 51.9, 33.4, 28.1, 25.7, 23.1, 18.4; **MS** (ESI) *m/z* (rel.%): 339 [MNa^+] (63), 317 [MH^+] (100), 210 (7), 180 (7); **HRMS** (ESI) calculated for $C_{18}H_{21}O_5$ [MH^+]: 317.1384, found: 317.1379; **IR** (neat, cm^{-1}): 3091, 2947, 2160, 1710, 1636, 1567, 1415, 1221, 1098, 823.

Methyl 3-(6-(2-phenylethyl)-2-pyronyl-4-oxy)-acrylate (7f)

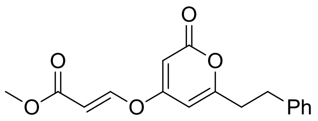

Synthesised using general procedure 2 and purified *via* flash column chromatography (20% EtOAc in hexanes) to afford a yellow crystalline powder (19.3 mg, 68%).

^{1}H-NMR (400 MHz, $CDCl_3$): 7.65 (d, *J* = 12.1 Hz, 1H), 7.33–7.28 (m, 2H), 7.25–7.20 (m, 1H), 7.19–7.15 (m, 2H), 5.84 (dt, *J* = 2.3, 0.7 Hz, 1H), 5.82 (d, *J* = 12.1 Hz, 1H), 5.66 (d, *J* = 2.3 Hz, 1H), 3.77 (s, 3H), 2.99 (t, *J* = 7.8 Hz, 2H), 2.81 (td, *J* = 7.7, 0.7 Hz, 2H); **^{13}C-NMR** (101 MHz, $CDCl_3$): 167.1, 166.5, 166.0, 163.5, 152.5, 139.6, 128.8, 128.4, 126.7, 107.8, 99.2, 92.5, 52.0, 35.8, 32.9; **MS** (ESI) *m/z* (rel. %): 301 [MH^+] (100); **HRMS** (ESI) calculated for $C_{17}H_{17}O_5$: 301.1071, found: 301.1069; **IR** ($CHCl_3$, cm^{-1}): 3019, 2363, 1721, 1641, 1573, 1416, 1101, 670.

Methyl 3-(6-(but-3-enyl)-2-pyronyl-4-oxy)-acrylate (7g)

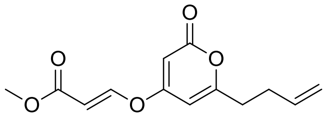

Synthesised using general procedure 2 and purified *via* flash column chromatography (20% EtOAc in hexanes) to afford a yellow crystalline powder (18.1 mg, 86%).

^{1}H-NMR (500 MHz, $CDCl_3$): 7.67 (d, *J* = 12.1 Hz, 1H), 5.90 (d, *J* = 2.1 Hz, 1H), 5.84 (d, *J* = 12.1 Hz, 1H), 5.78 (ddt, *J* = 17.0, 10.1, 6.7 Hz, 1H), 5.65 (d, *J* = 2.1 Hz, 1H), 5.08 (ddt, *J* = 17.0, 1.5, 1.5 Hz, 1H), 5.05 (ddt, *J* = 10.1, 1.5, 1.5 Hz, 1H), 3.77 (s, 3H), 2.60 (t, *J* = 7.9 Hz, 2H), 2.41–2.45 (m, 2H); **^{13}C-NMR** (101 MHz, $CDCl_3$): 167.1, 166.8, 165.9, 163.3, 152.5, 135.7, 116.6, 107.7, 98.8, 92.3, 51.8, 33.2, 30.5; **MS** (ESI) *m/z* (rel.%): 251 [MH^+] (100); **HRMS** (ESI) calculated for $C_{13}H_{15}O_5$: 251.0914, found: 251.0917; **IR** ($CHCl_3$, cm^{-1}): 3077, 2955, 2362, 1722, 1639, 1574, 1415, 1325, 1223, 1163, 1099.

Methyl 3-(6-(6-triisopropylsilyl-hex-5-ynyl)-2-pyronyl-4-oxy)-acrylate (7h)

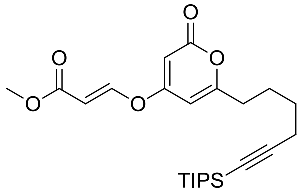

Synthesised using general procedure 2 and purified *via* flash column chromatography (20% EtOAc in hexanes) to afford a waxy yellow powder (102 mg, 82%).

^{1}H-NMR (400 MHz, $CDCl_3$): 7.68 (d, *J* = 12.0 Hz, 1H), 5.90 (d, *J* = 2.3 Hz, 1H), 5.84 (d, *J* = 12.0 Hz, 1H), 5.65 (d, *J* = 2.3 Hz, 1H), 3.77 (s, 3H), 2.53 (t, *J* = 7.5 Hz, 2H), 2.30 (t, *J* = 6.9 Hz, 2H), 1.77–1.86 (m, 2H), 1.54–1.63 (m, 2H), 1.00–1.08 (m, 21H); **^{13}C-NMR** (101 MHz, $CDCl_3$): 167.5, 167.1, 165.9, 163.4, 152.4, 107.8, 107.7, 98.5, 92.1, 81.0, 51.8, 33.2, 27.8, 25.5, 19.4, 18.6, 11.2; **MS** (ESI) *m/z* (rel.%): 455 [MNa^+] (100), 433 [MH^+] (51), 399 (5), 377 (3), 326 (4), 289 (12), 267 (8), 242 (10), 217 (7), 180 (4); **HRMS** (ESI) calculated for $C_{24}H_{36}NaO_5Si$ [MNa^+]: 455.2224, found: 455.2213; **IR** ($CHCl_3$, cm^{-1}): 3023, 2956, 2866, 2362, 1721, 1641, 1574, 1418, 1215, 1101.

Pentafluorophenyl 3-(6-(6-triisopropylsilyl-hex-5-ynyl)-2-pyronyl-4-oxy)-acrylate (7i)

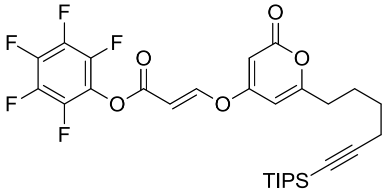

Synthesised using general procedure 2 and product purified *via* flash column chromatography (20% EtOAc in hexanes) to afford a waxy yellow powder (65.7 mg, 37%).

^{1}H-NMR (400 MHz, $CDCl_3$): 7.94 (d, *J* = 12.0 Hz, 1H), 6.06 (d, *J* = 12.0 Hz, 1H), 5.97 (d, *J* = 2.3 Hz, 1H), 5.75 (d, *J* = 2.3 Hz, 1H), 2.57 (t, *J* = 7.6 Hz, 2H), 2.32 (t, *J* = 6.9 Hz, 2H), 1.79–1.89 (m, 2H), 1.55–1.66 (m, 2H), 1.01–1.09 (m, 21H); **^{13}C-NMR** (101 MHz, $CDCl_3$): 168.1, 166.8, 163.2, 161.5, 155.8, 107.8, 104.3, 98.3, 93.0, 81.1, 33.3, 27.8, 25.5, 19.4, 18.6, 11.2 (carbons on perfluorophenyl ring not observed); **^{19}F-NMR** (376 MHz, $CDCl_3$): −152.22–−152.50 (m, 2F), −157.29 (t, *J* = 21.7, 1F), −161.75–−162.06 (m, 2F); **MS** (ESI) *m/z* (rel. %): 607 [MNa^+] (79), 471 (53), 284 (74), 247 (100), 225 (20); **HRMS** (ESI) calculated for $C_{29}H_{33}F_5NaO_5Si$ [MNa^+]: 607.1910, found: 607.1925; **IR** (neat, cm^{-1}): 3093, 2946, 2865, 2167, 1717, 1637, 1571, 1517, 1181, 1005.

Ethyl 3-(6-methyl-2-pyronyl-4-oxy)-but-2-enoate (9)

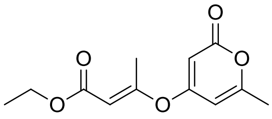

Method A: Synthesised using general procedure 2 with allene **8** and purified *via* flash column chromatography (30% EtOAc in hexanes) to afford a waxy yellow solid (61.4 mg, 52%).

Method B: A solution of 4-hydroxy-6-methyl-2-pyrone (**3a**) (126 mg, 1 mmol, 1 eq.), ethyl 2-butynoate (134 mg, 1.2 mmol, 1.2 eq.), DBU (100 mg, 0.66 mmol, 0.66 eq.), and CuI (19 mg, 0.1 mmol, 10 mol%) in THF (2 mL) was heated under nitrogen at 80 °C in a microwave for 30 minutes. The solvent was removed *in vacuo* and the product purified by column chromatography (30% EtOAc in hexanes) to afford a waxy yellow solid (102 mg, 43%).

R_f = 0.43 (40% EtOAc in hexanes); **^{1}H-NMR** (400 MHz, $CDCl_3$): 5.87 (dq, *J* = 2.0, 0.8 Hz, 1H), 5.57 (dd, *J* = 2.0, 0.6 Hz, 1H), 5.52 (q, *J* = 0.9 Hz, 1H), 4.19 (q, *J* = 7.1 Hz, 2H), 2.38 (d, *J* = 0.9 Hz, 3H), 2.26 (dd, *J* = 0.8, 0.6 Hz, 3H), 1.29 (t, *J* = 7.1 Hz, 3H); **^{13}C-NMR** (100 MHz, $CDCl_3$): 167.2, 165.8, 165.6, 163.9, 107.5, 100.1, 94.4, 79.9, 60.4, 20.1, 17.4, 14.2; **MS** (ESI) *m/z* (rel. %): 261 [MNa^+] (100), 177 (2), 153 (4); **HRMS** (ESI) calculated for

$C_{12}H_{14}O_5Na$ [M^+]: 261.0733, found: 261.0738; **IR** (CH_2Cl_2, cm^{-1}): 3018, 2919, 2871, 2774, 1729, 1643, 1556, 1384, 1335, 1168.

5. References

1. Burns, M. J.; Thatcher, R. J.; Taylor, R. J. K.; Fairlamb, I. J. S. *Dalton Trans.* **2010,** *39*, 10391–10400.
2. Pàmies, O.; Bäckvall, J.-E. *J. Org. Chem.* **2003,** *68*, 4815–4818.
3. Wong, C. H.; Mazenod, F. P.; Whitesides, G. M. *J. Org. Chem.* **1983,** *48*, 3493–3497.
4. Alexakis, A.; Cahiez, G.; Normant, J. F. *Synthesis* **1979,** *1979*, 826–830.
5. Pirrung, F. O. H.; Hiemstra, H.; Nico Speckamp, W.; Kaptein, B.; Schoemaker, H. E. *Tetrahedron* **1994,** *50*, 12415–12442.
6. Brooke, G. M.; Matthews, R. S.; Harman, M. E.; Hursthouse, M. B. *J. Fluorine Chem.* **1991,** *53*, 339–354.
7. Zhang, X.; McLaughlin, M.; Muñoz, R. L. P.; Hsung, R. P.; Wang, J.; Swidorski, J. *Synthesis* **2007,** *2007*, 749–753.
8. Begley, M. J.; Pattenden, G.; Robertson, G. M. *J. Chem. Soc., Perkin Trans. 1* **1988**, 1085–1094.
9. Shimo, T.; Yasuda, M.; Tajima, J.; Somekawa, K. *J. Heterocyclic Chem.* **1991,** *28*, 745–748.
10. Bonini, C.; Federici, C.; Rossi, L.; Righi, G. *J. Org. Chem.* **1995,** *60*, 4803–4812.

6. Representative ^{1}H NMR spectra for novel compounds

The following ^{1}H NMR spectra are representative of the novel compounds synthesized in this study. Due to the polarity and/or volatility of some of these compounds, trace impurities (*e.g.* solvent) are visible in some cases.

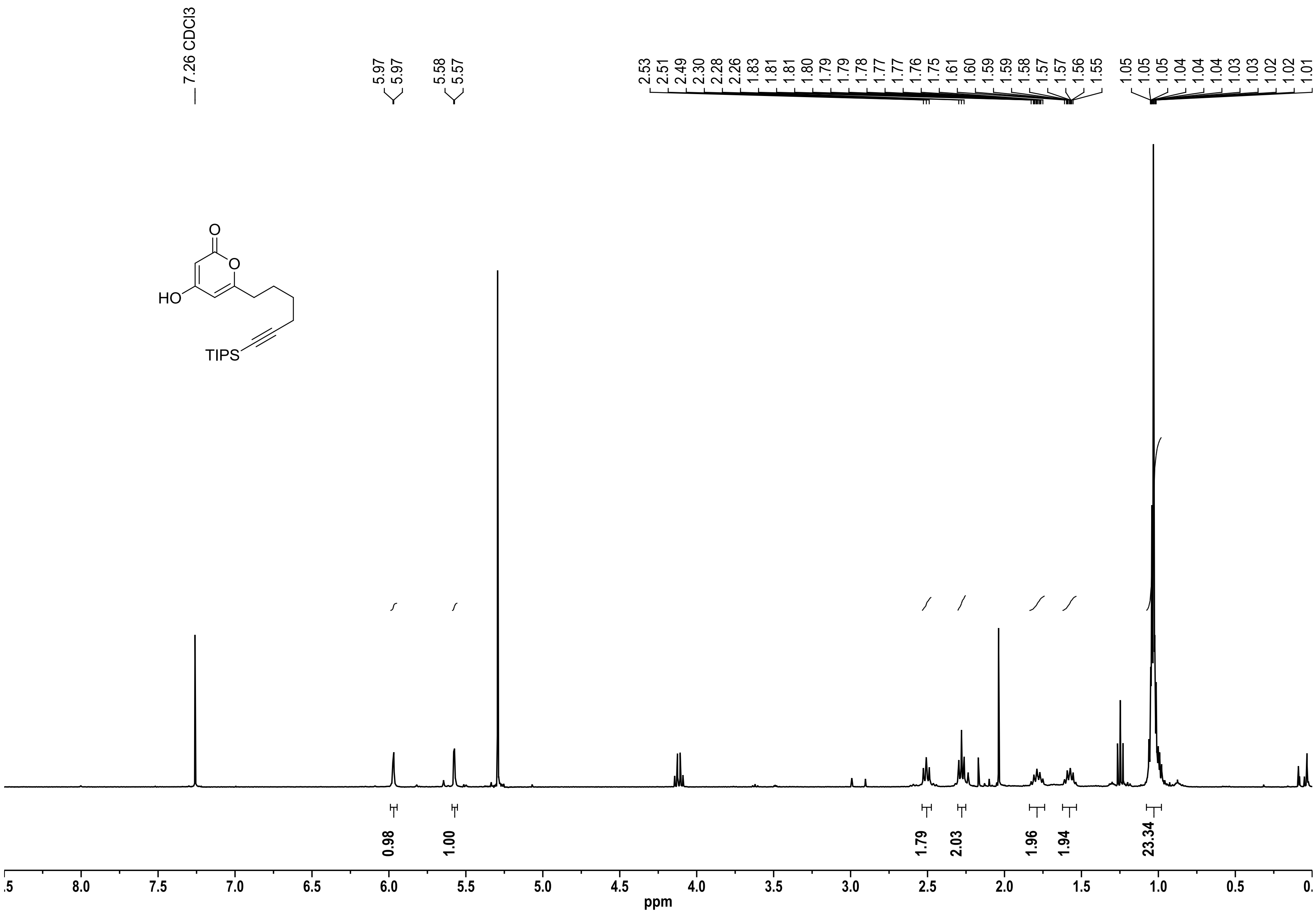

Figure S1 ^{1}H NMR spectrum of **3c**.

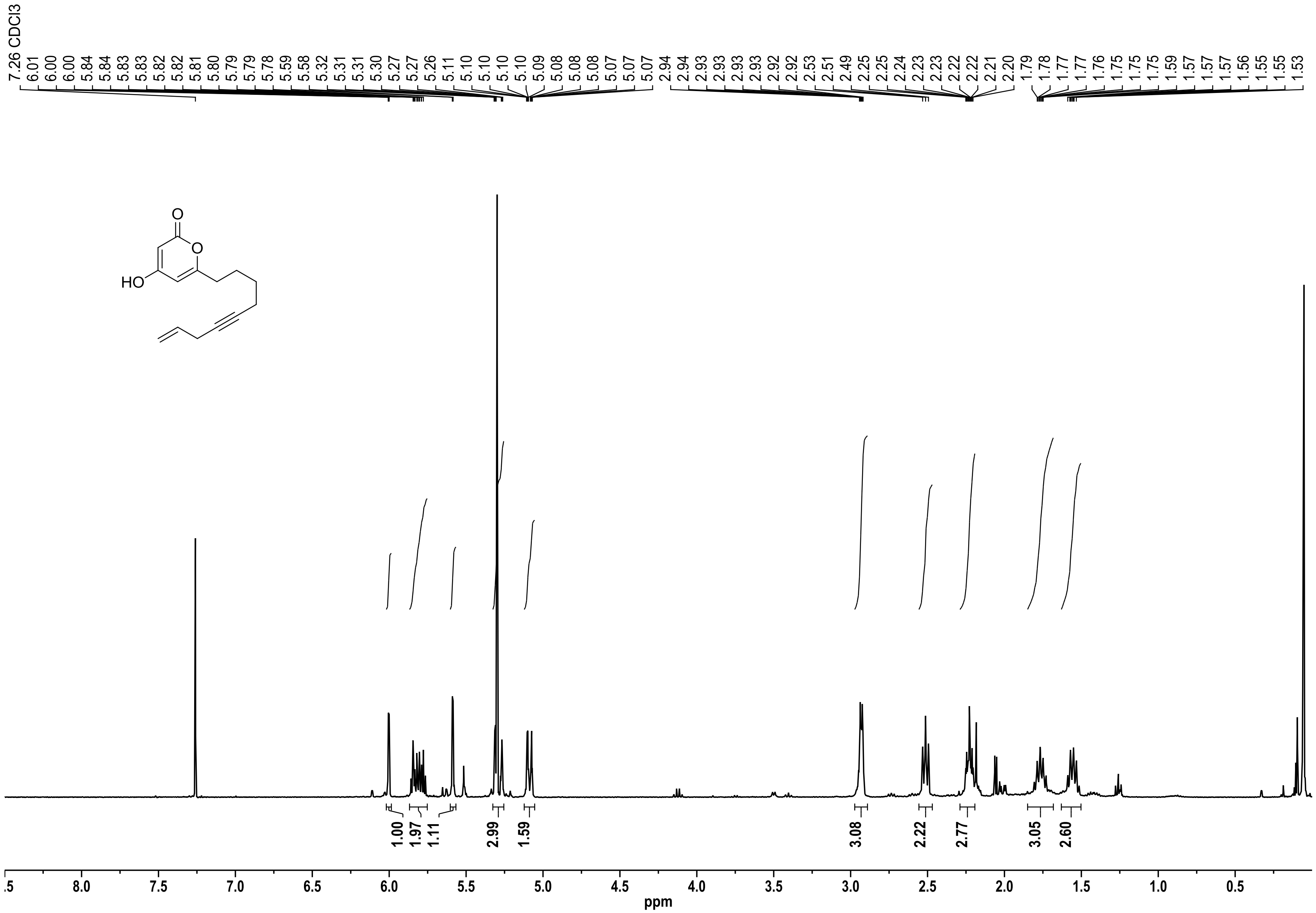

Figure S2 ^{1}H NMR spectrum of **3d**.

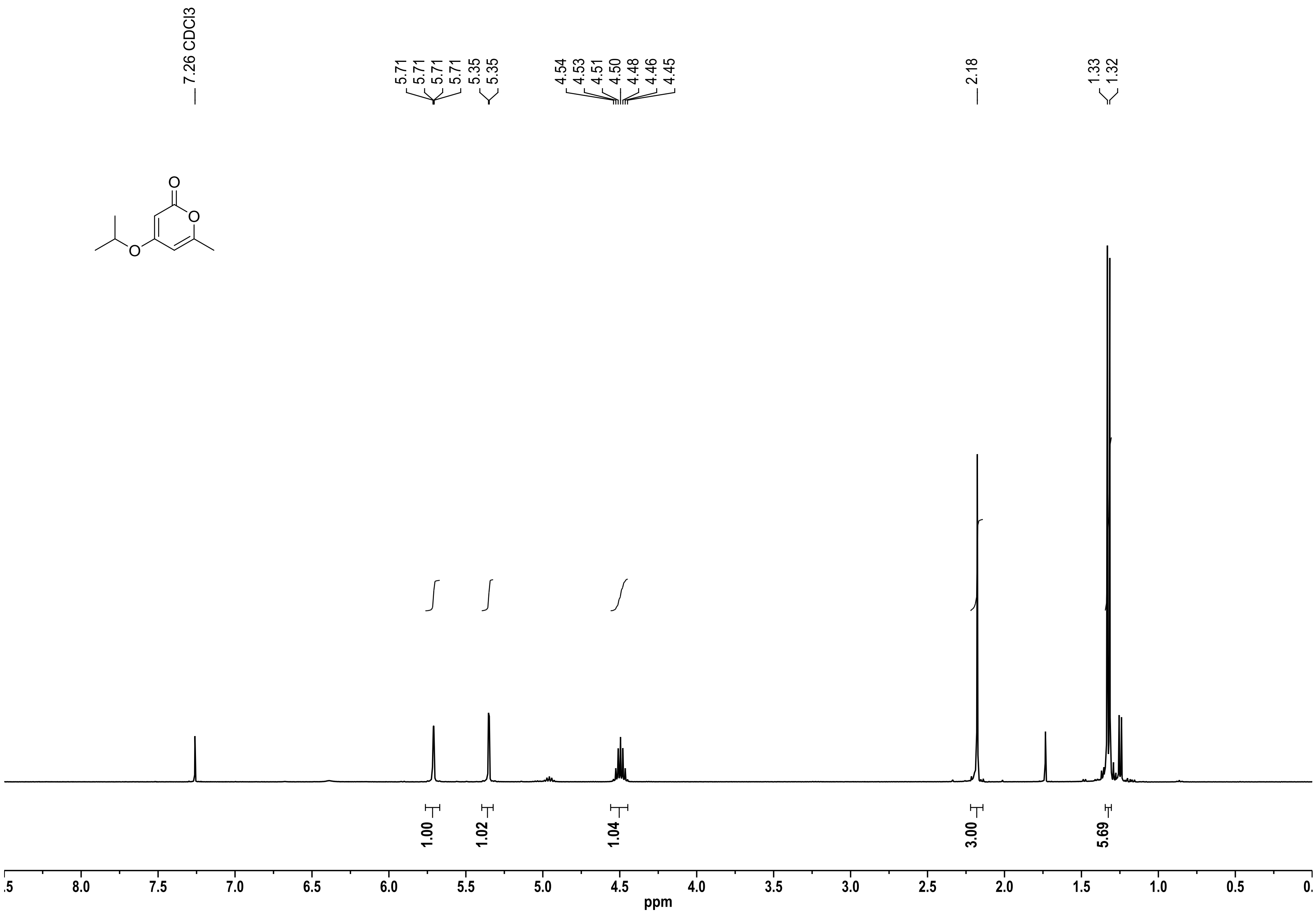

Figure S3 ^{1}H NMR spectrum of **4a**.

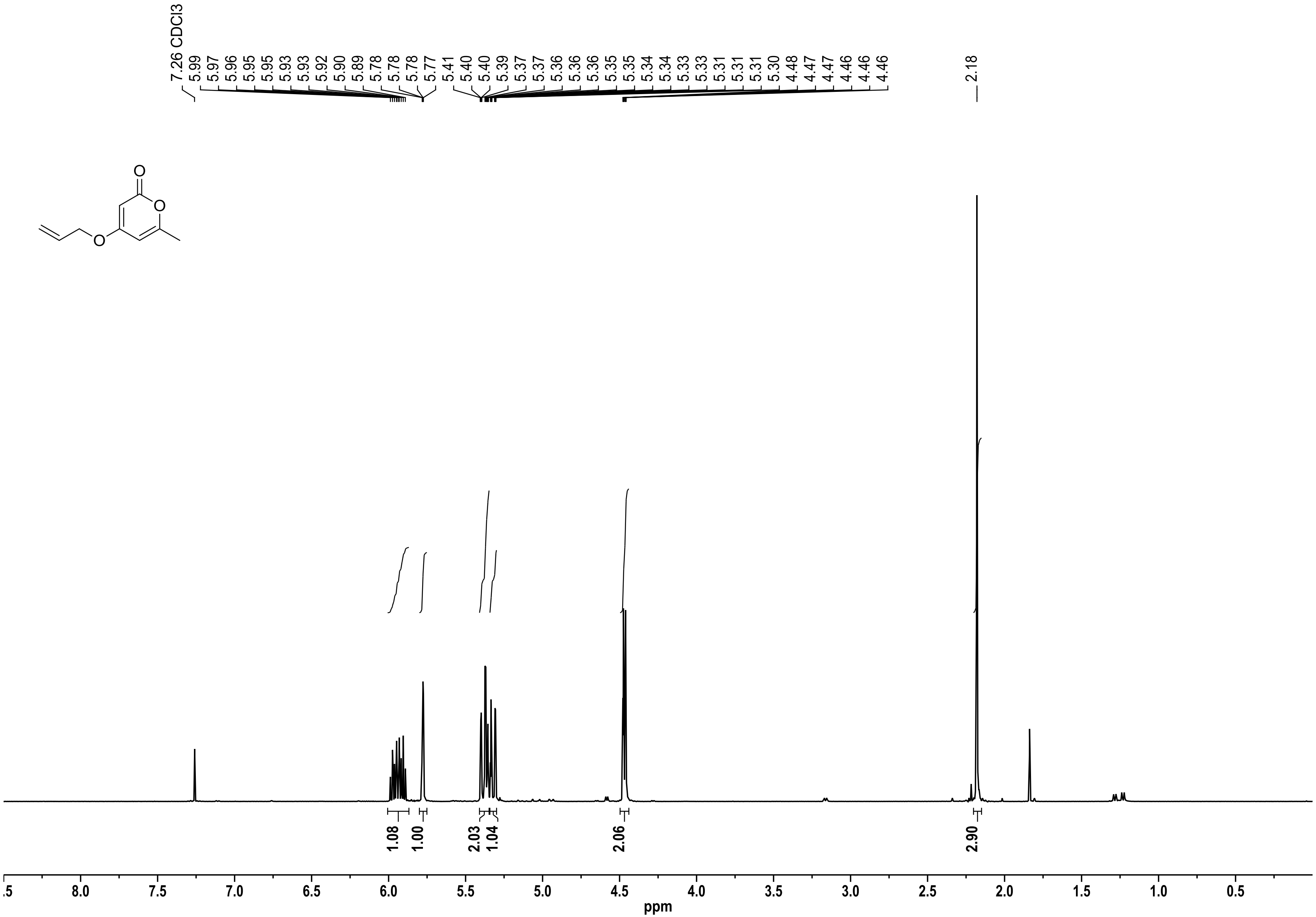

Figure S4 ^{1}H NMR spectrum of **4b**.

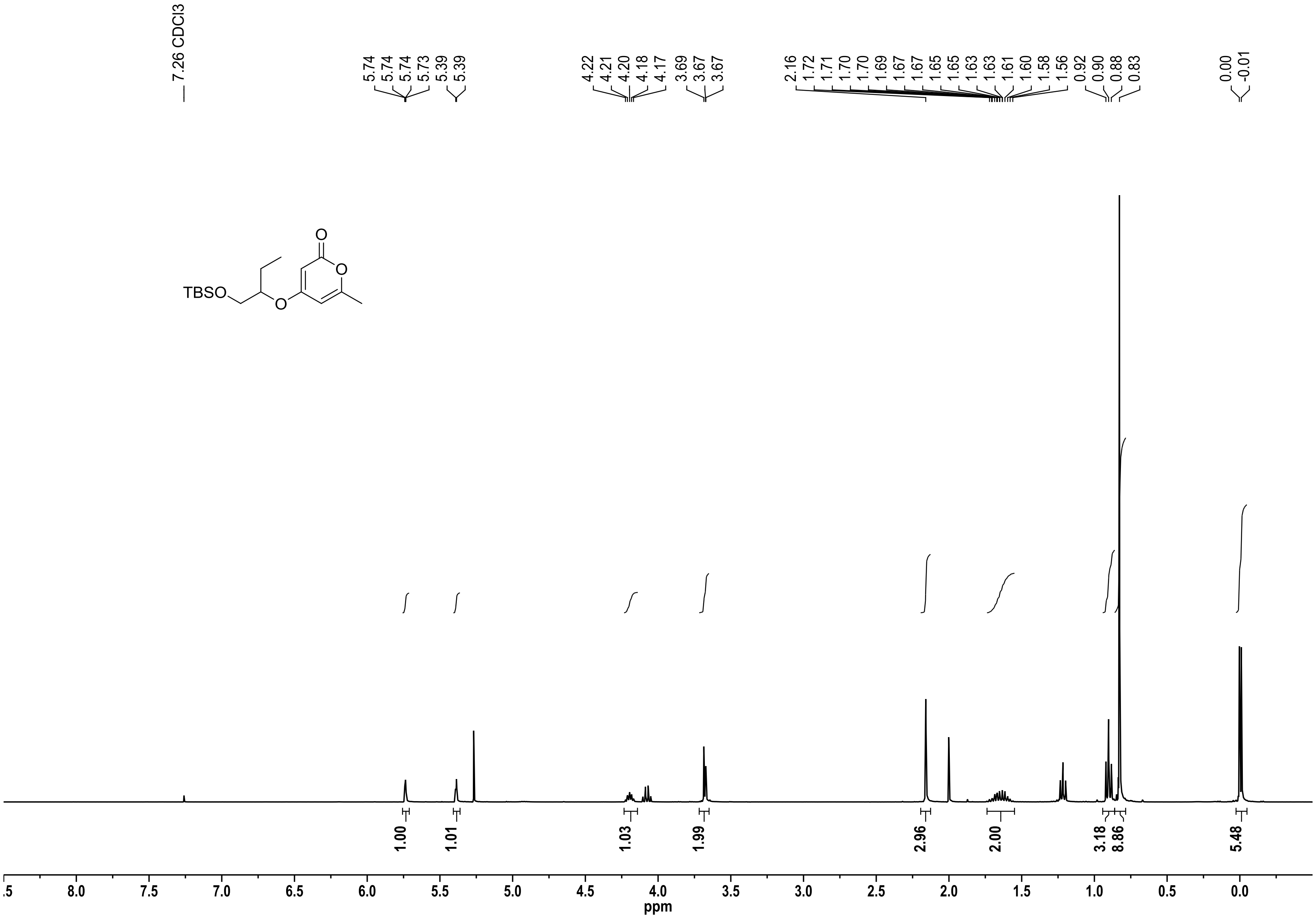

Figure S5 ^{1}H NMR spectrum of **4c**.

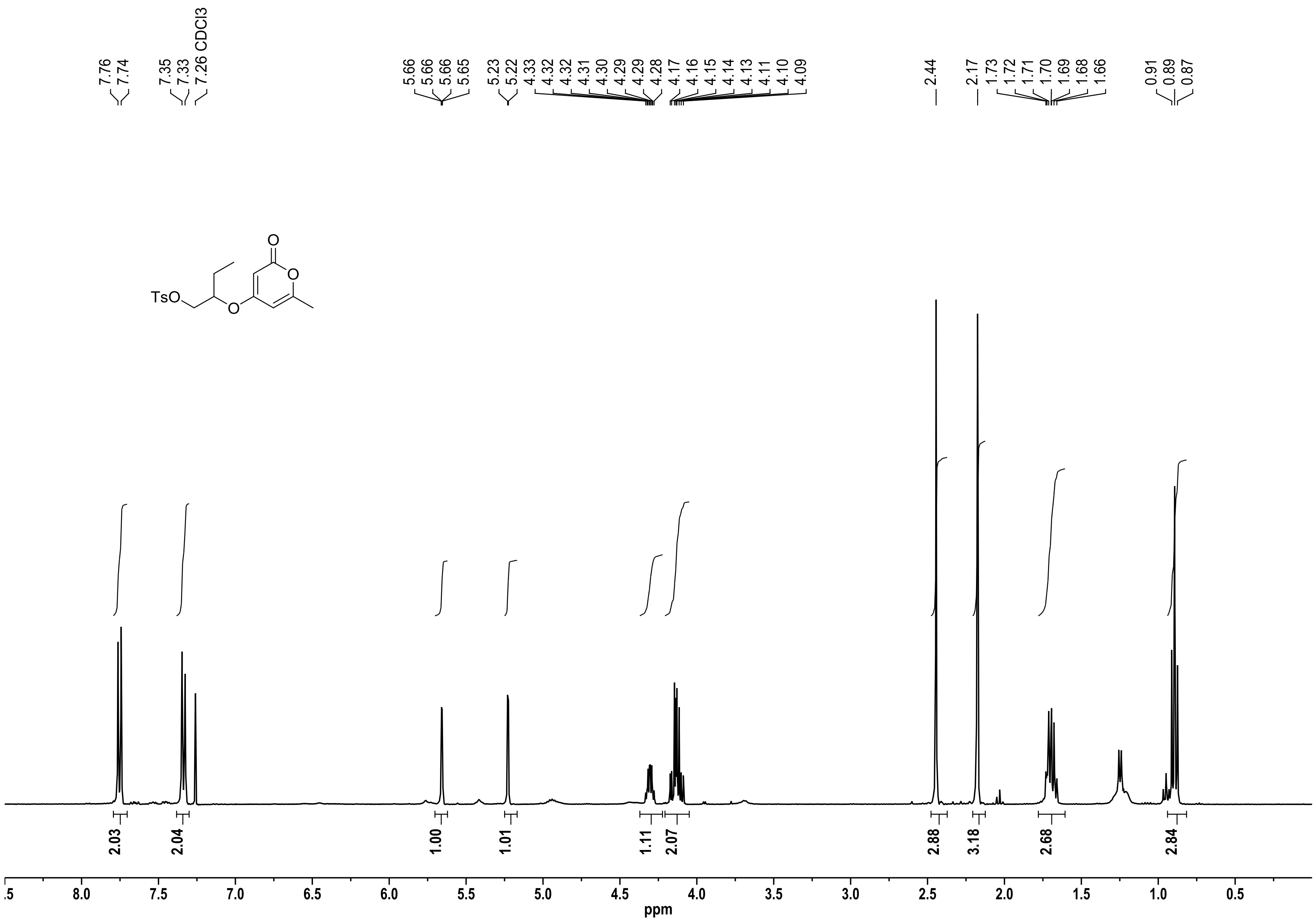

Figure S6 ^{1}H NMR spectrum of **4d**.

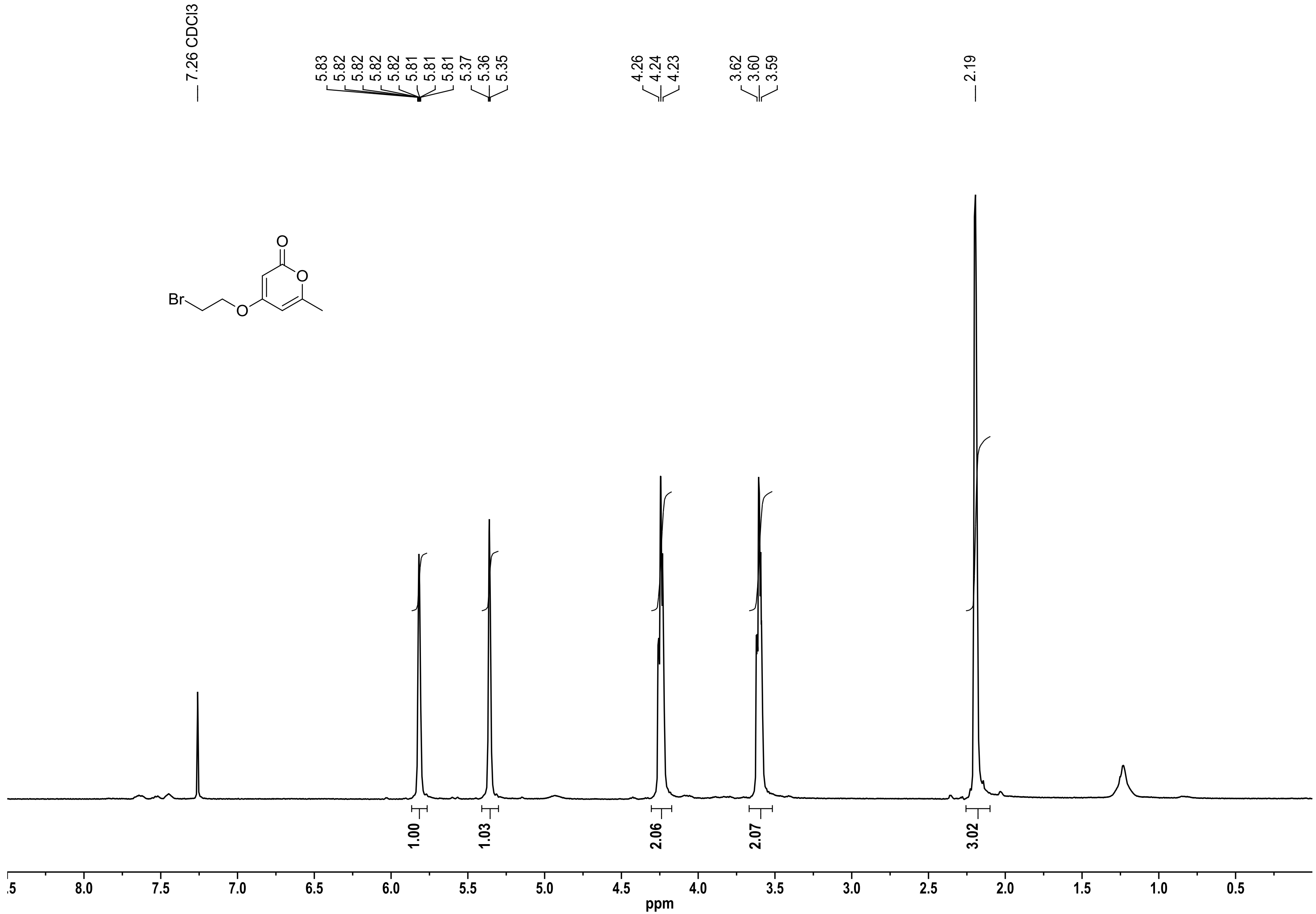

Figure S7 ^{1}H NMR spectrum of **4e**.

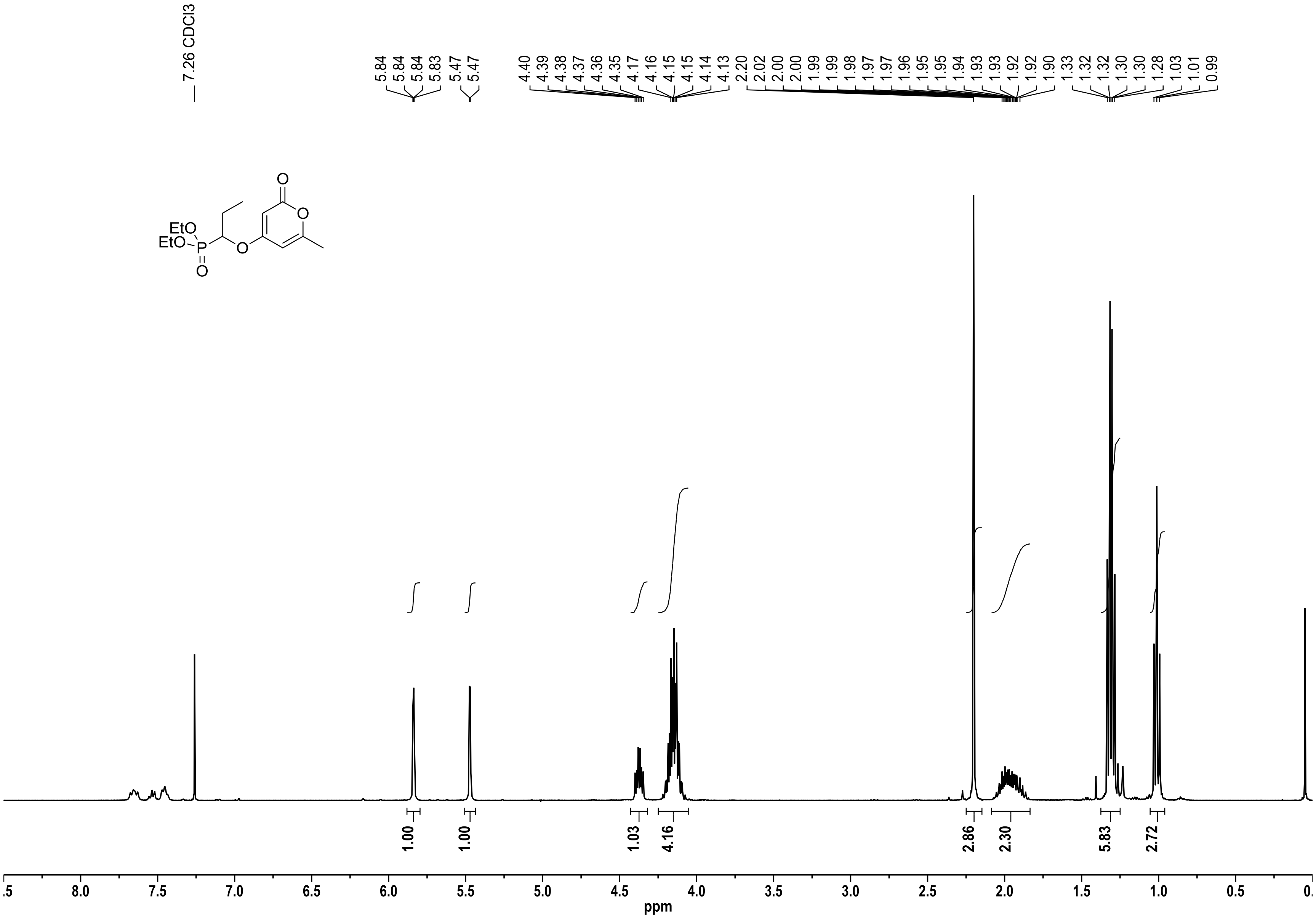

Figure S8 ^{1}H NMR spectrum of **4f**.

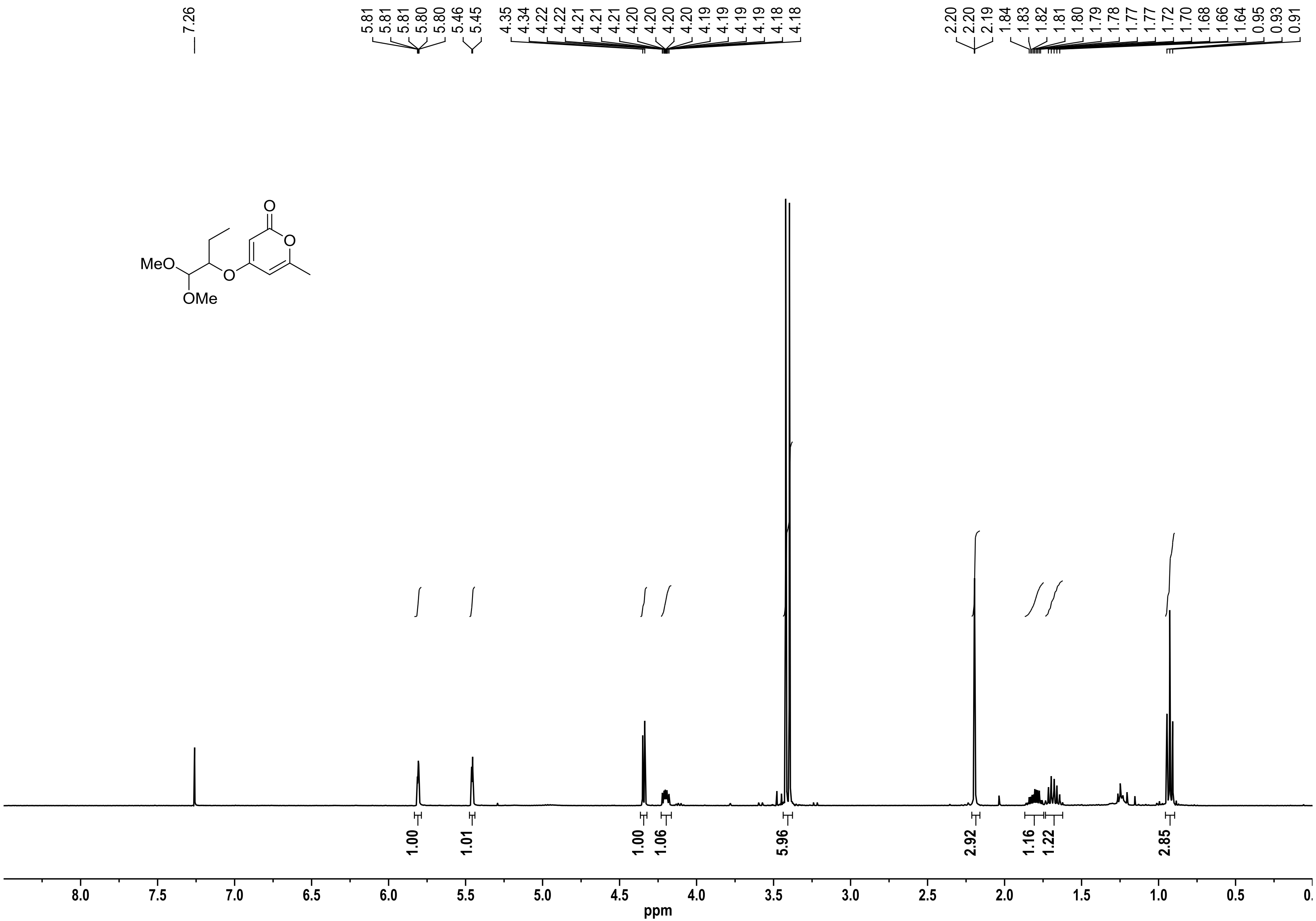

Figure S9 ^{1}H NMR spectrum of **4g**.

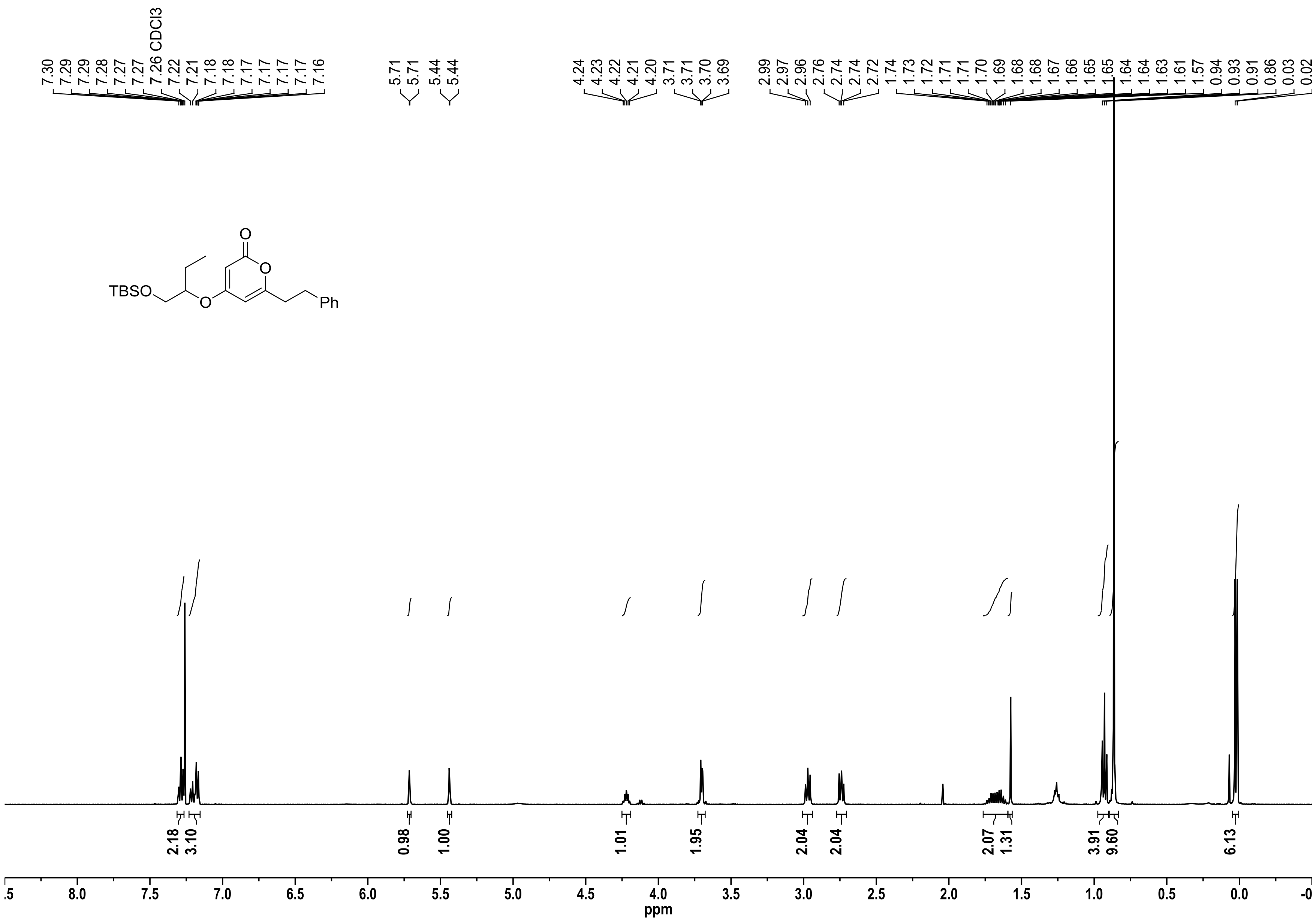

Figure S10 ^{1}H NMR spectrum of **4h**.

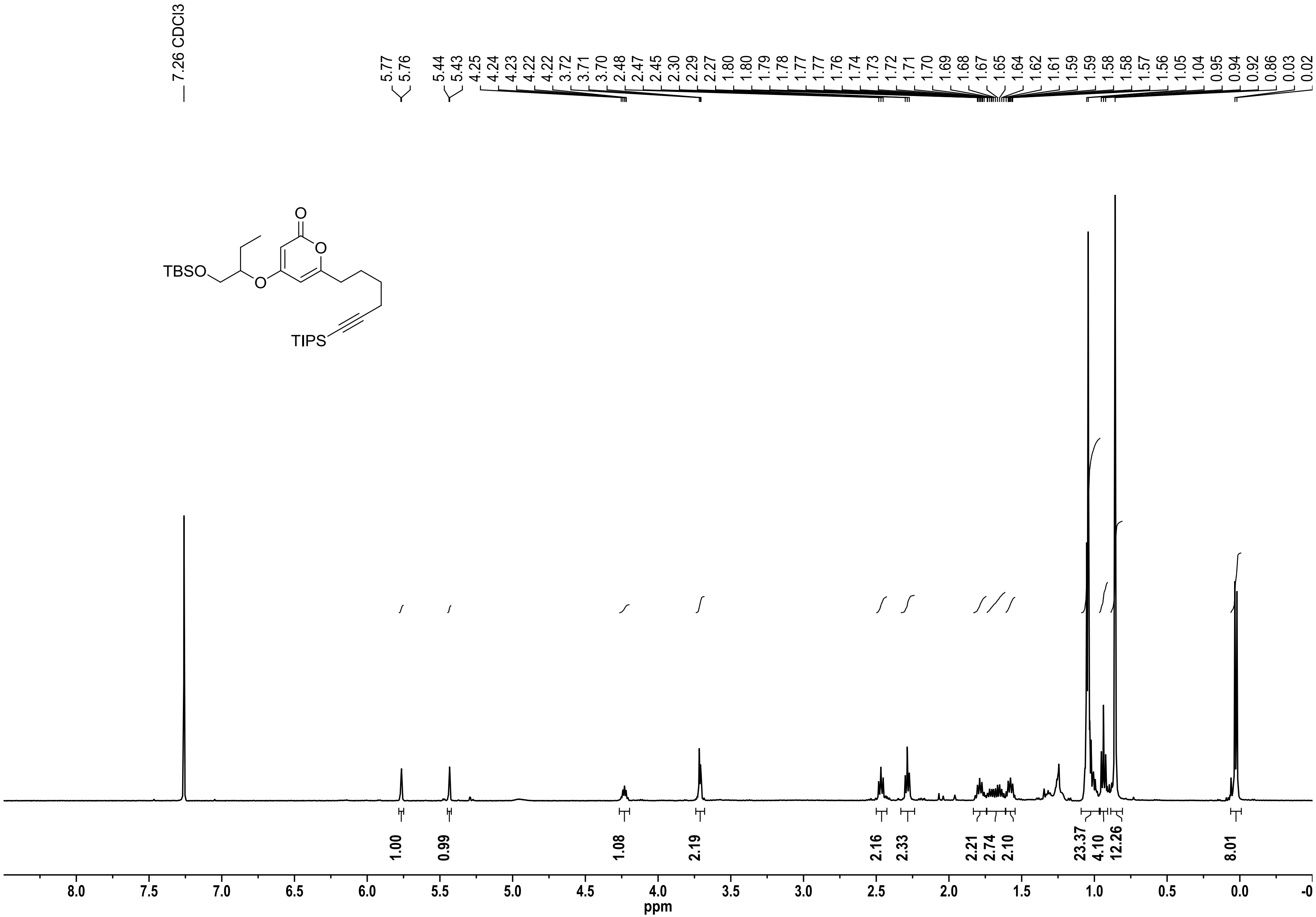

Figure S11 ^{1}H NMR spectrum of **4i**.

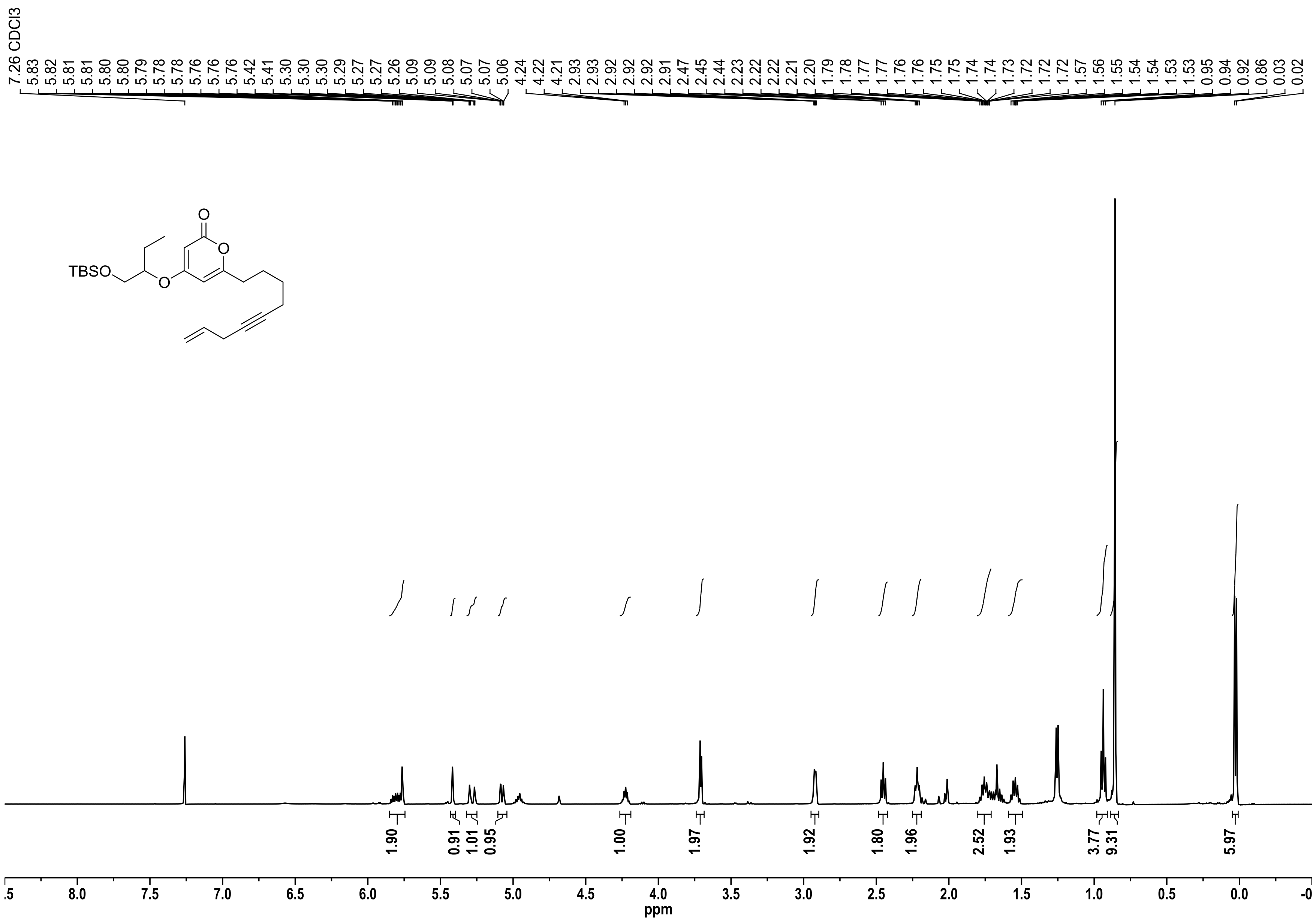

Figure S12 ^{1}H NMR spectrum of **4j**.

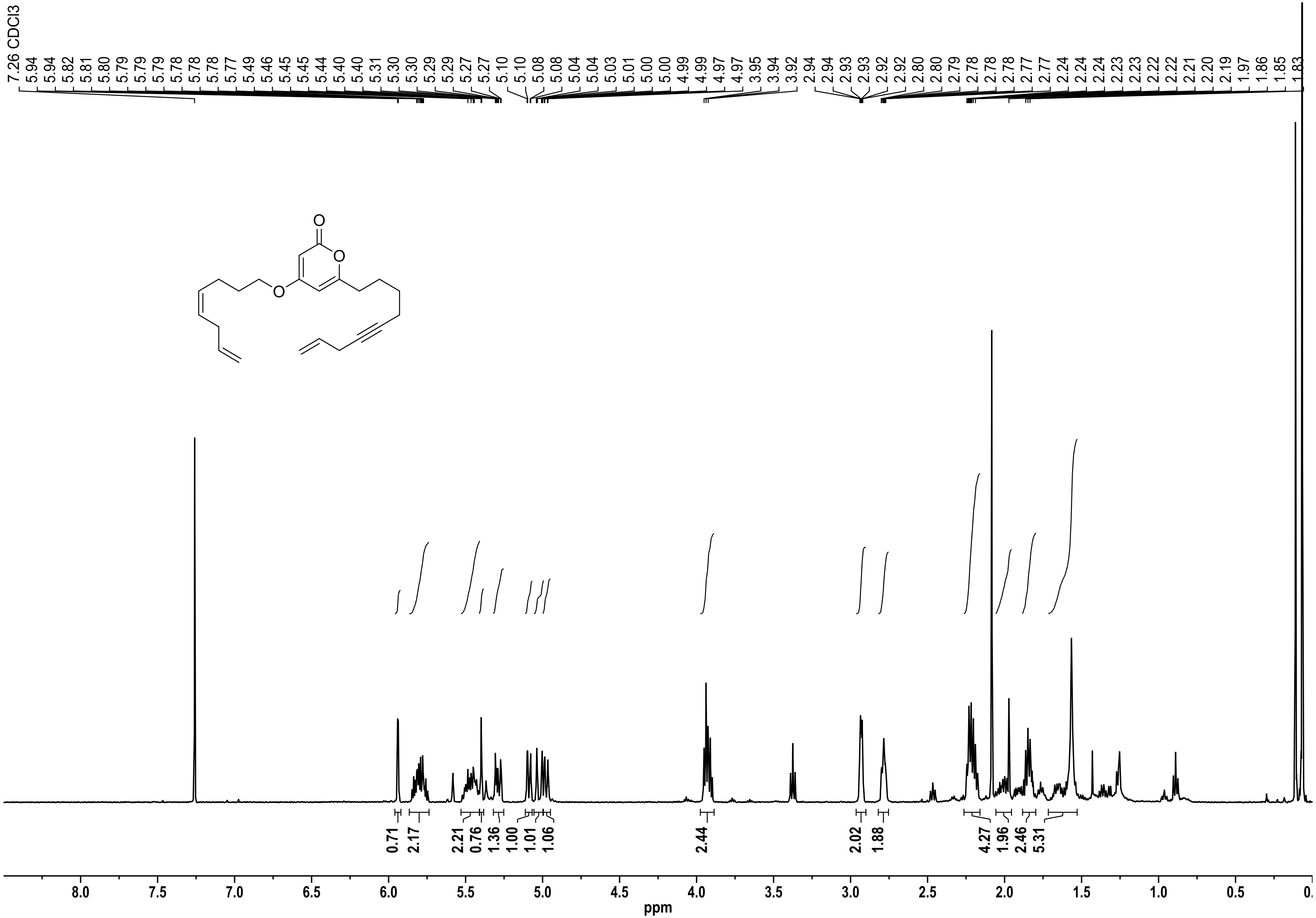

Figure S13 ^{1}H NMR spectrum of **4k**.

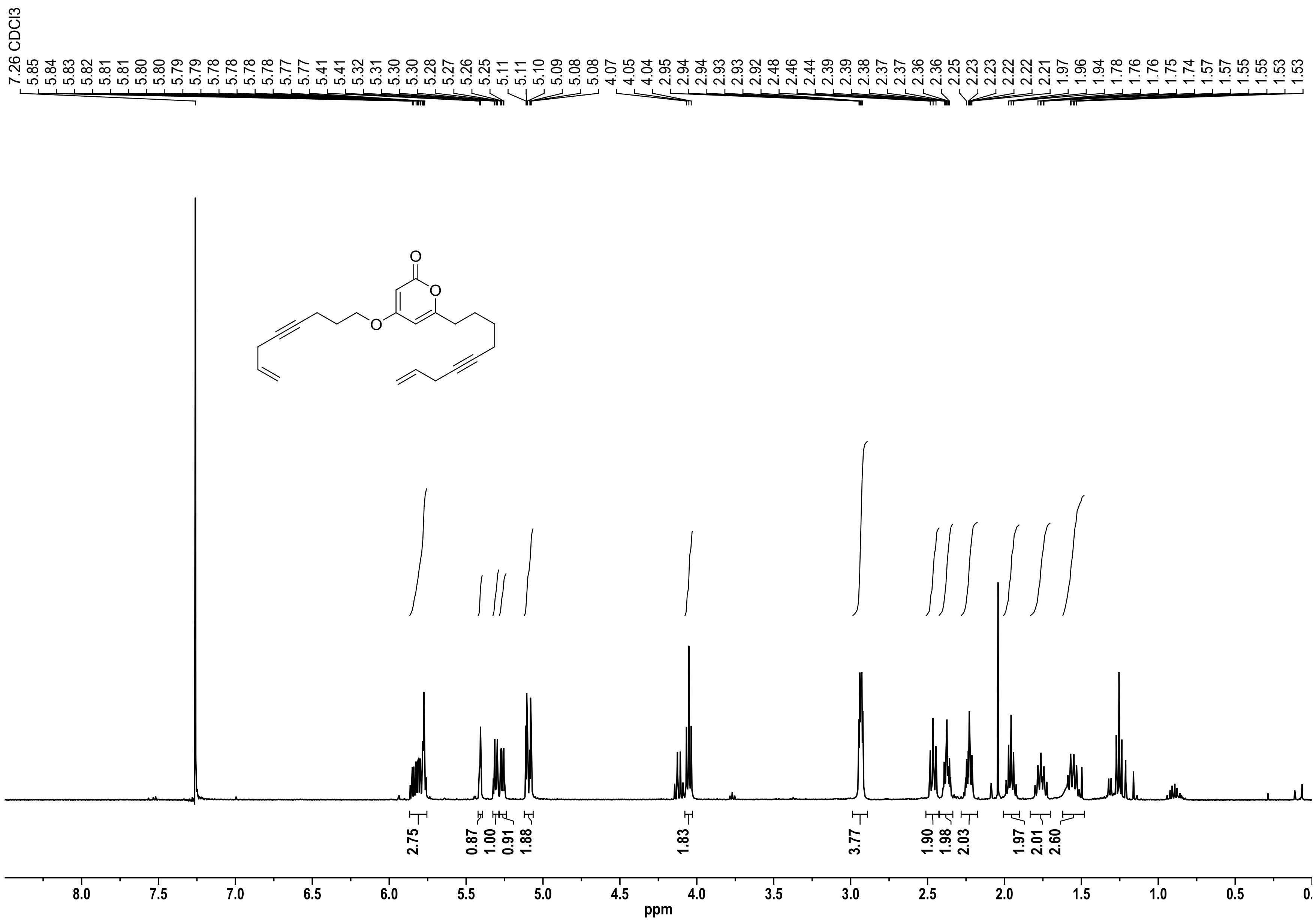

Figure S14 ^{1}H NMR spectrum of **4l**.

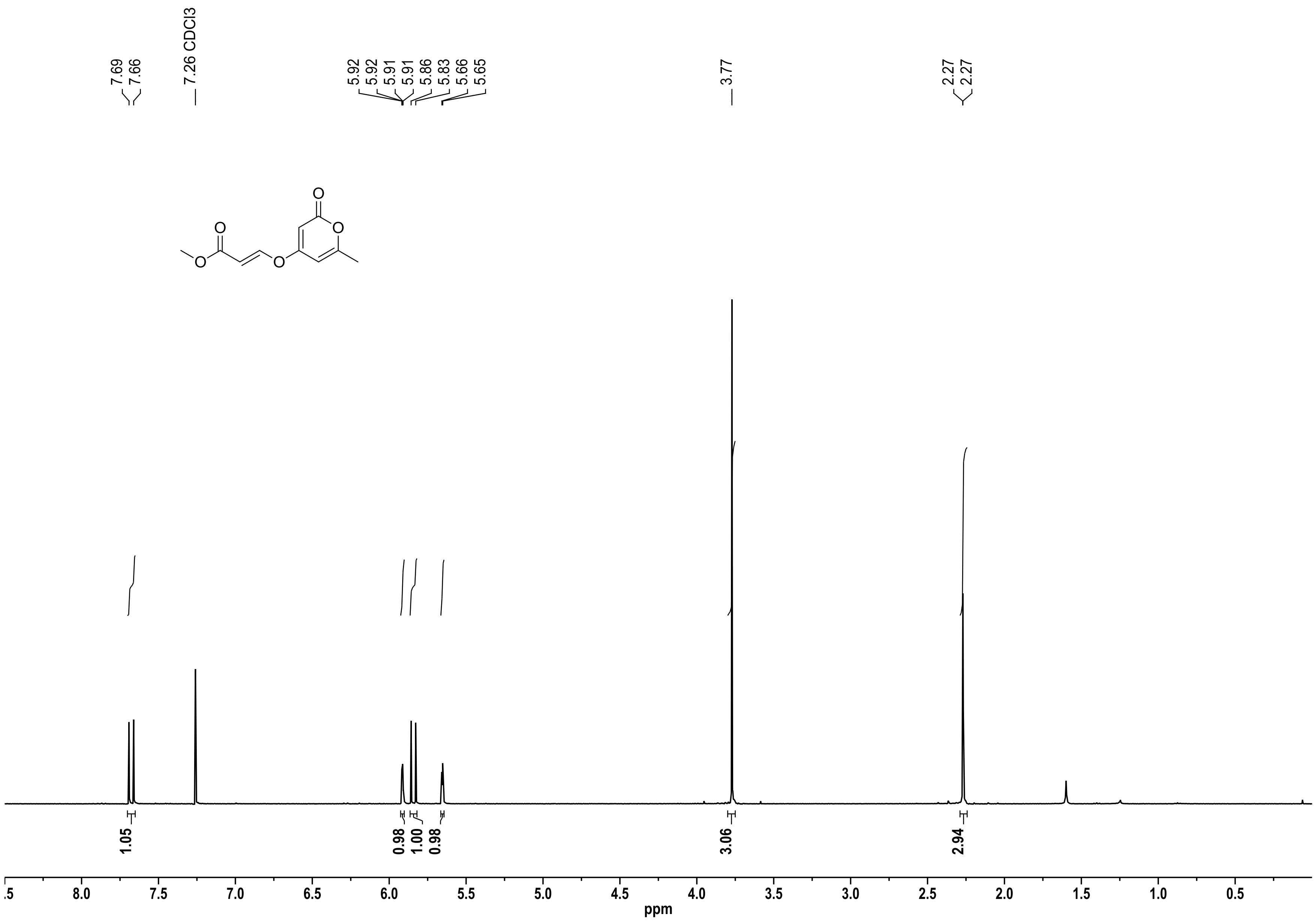

Figure S15 ^{1}H NMR spectrum of **7a**.

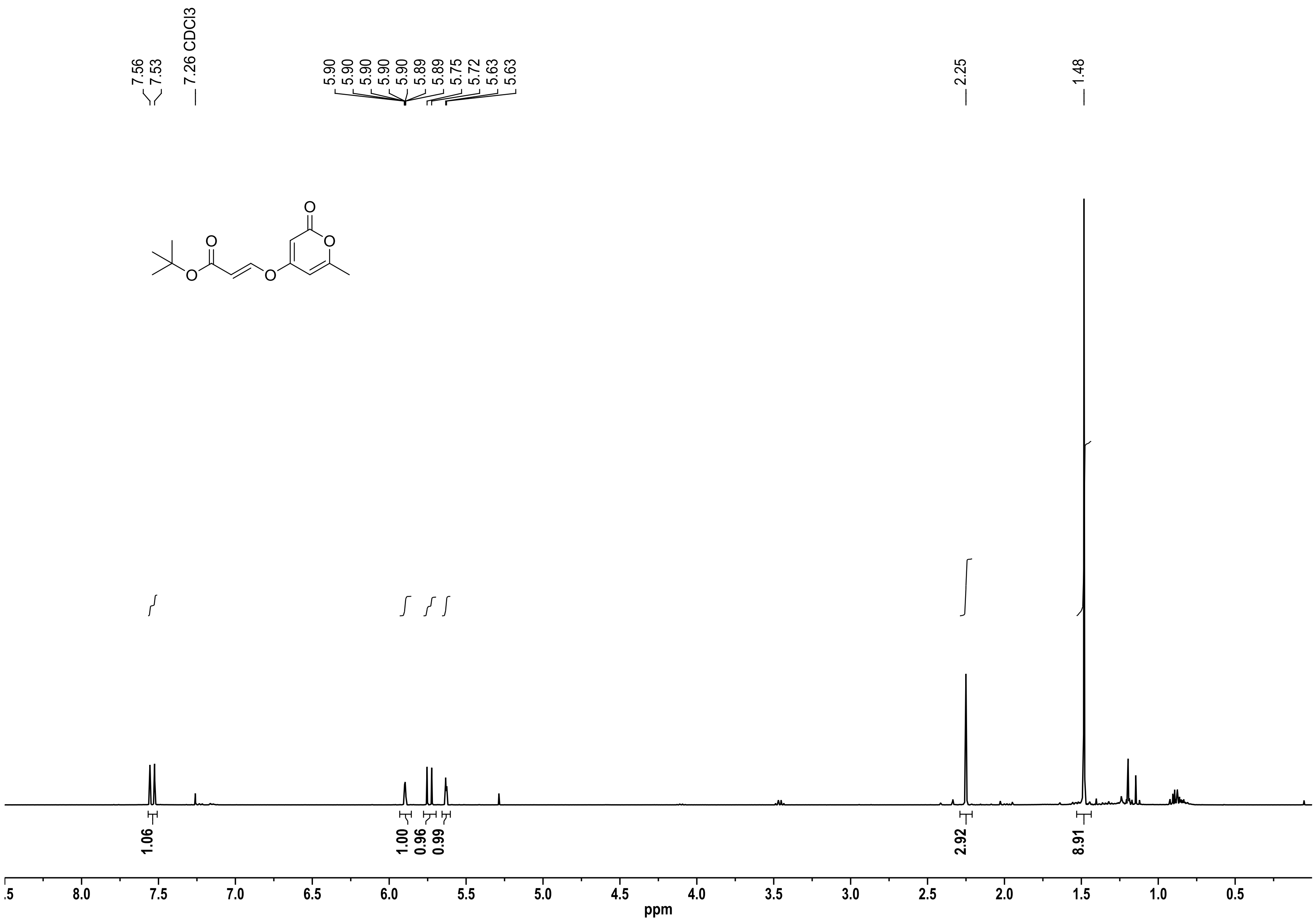

Figure S16 ^{1}H NMR spectrum of **7b**.

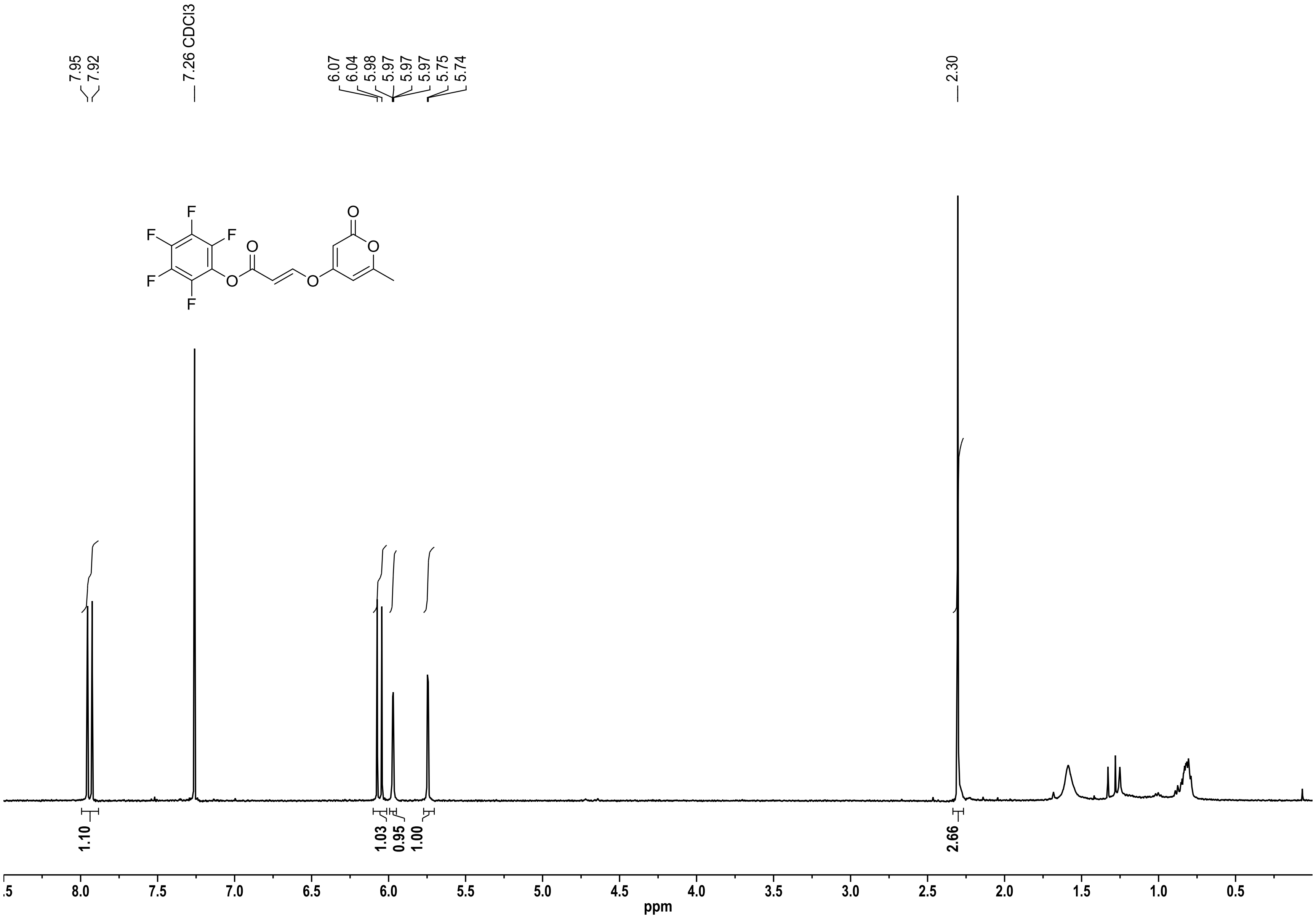

Figure S17 ^{1}H NMR spectrum of **7c**.

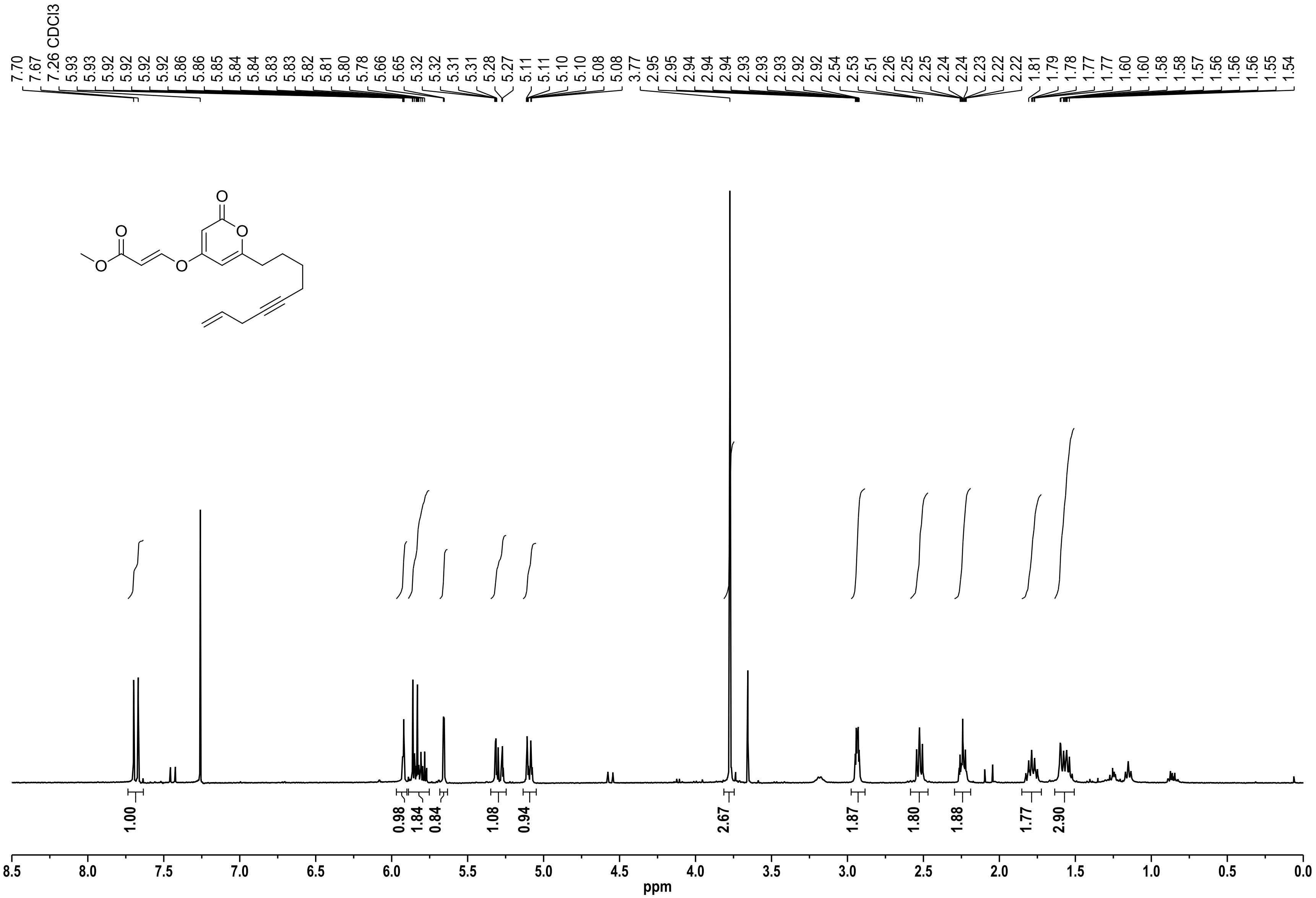

Figure S18 ^{1}H NMR spectrum of **7e**.

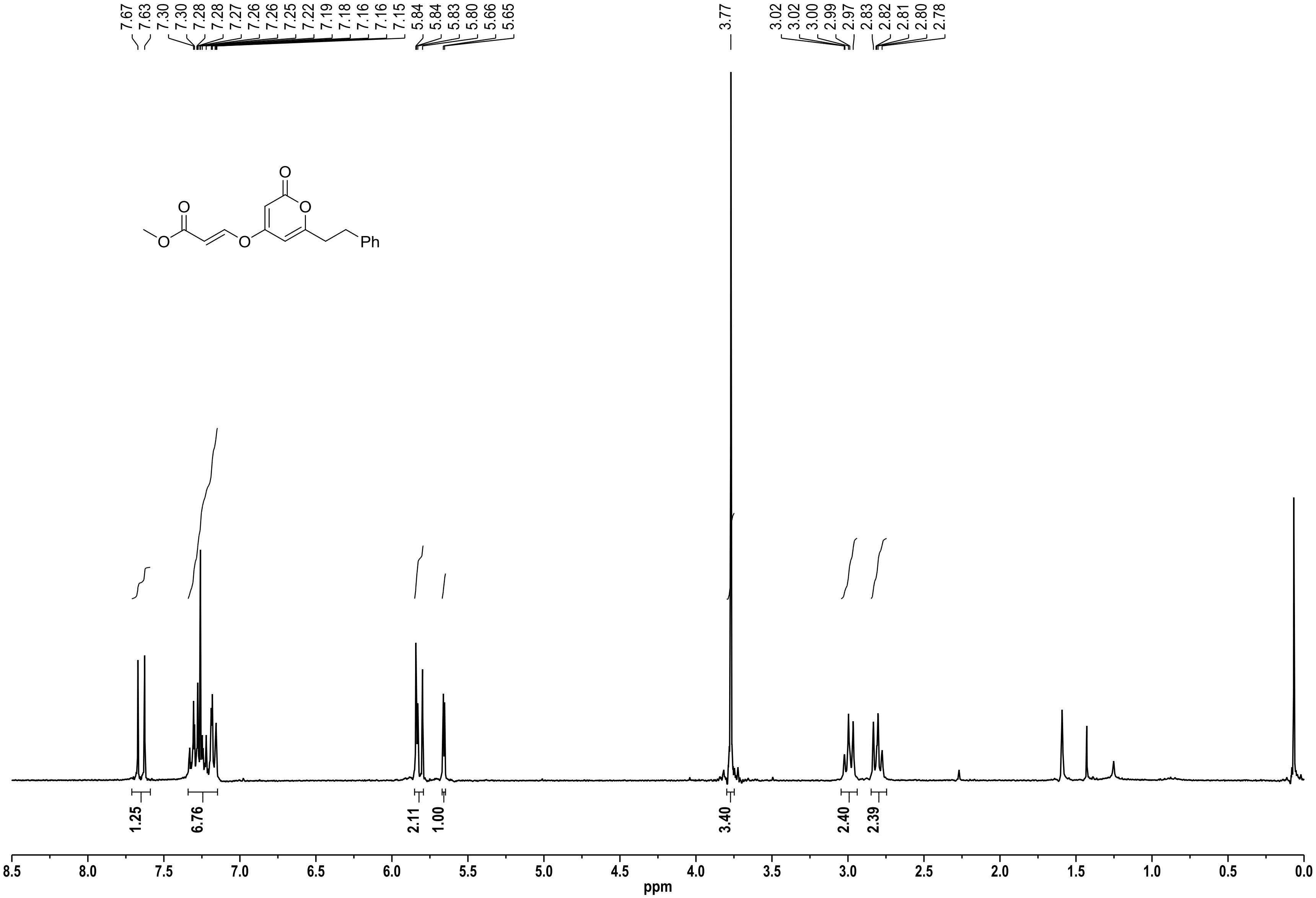

Figure S19 ^{1}H NMR spectrum of **7f.**

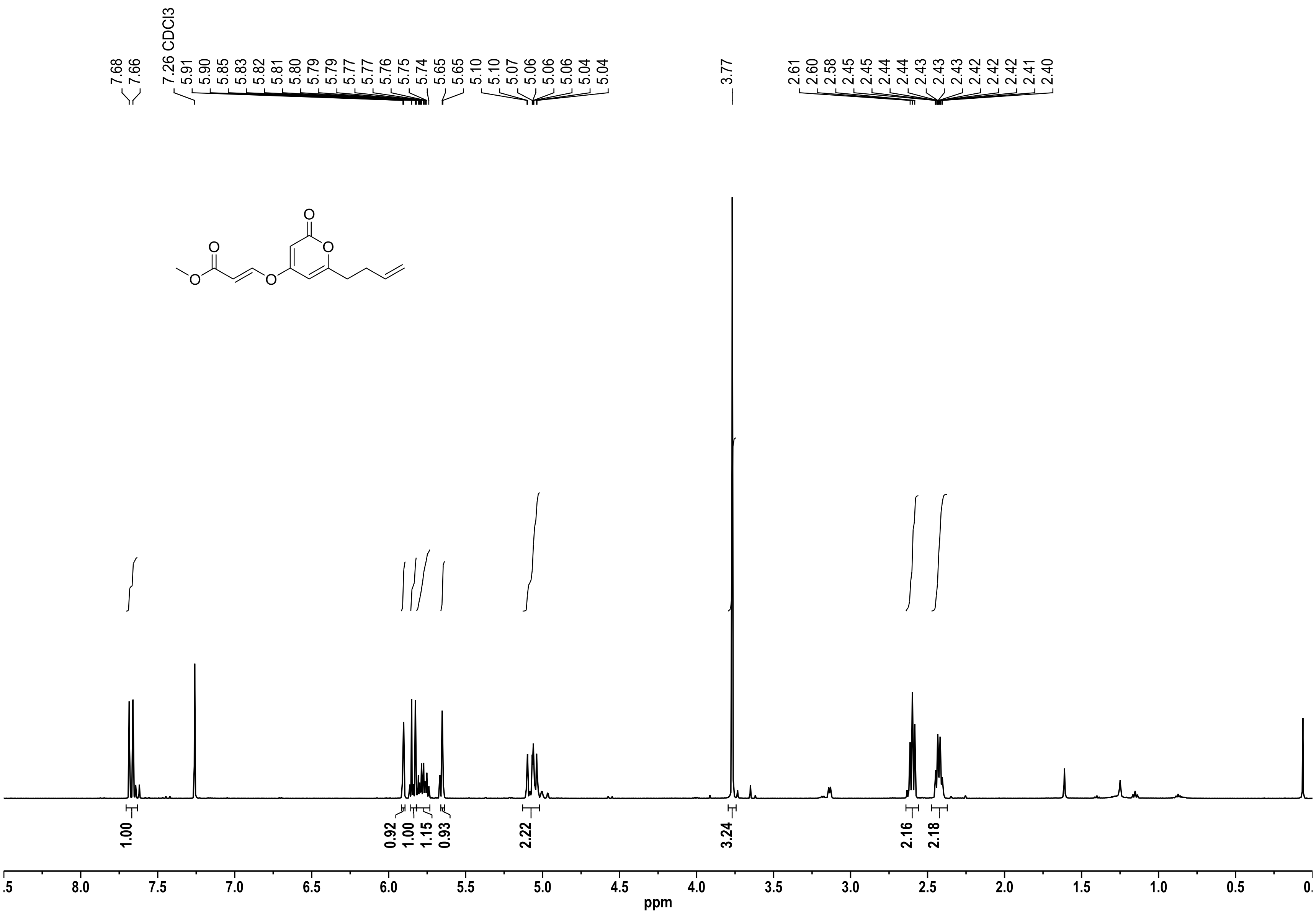

Figure S20 ^{1}H NMR spectrum of **7g**.

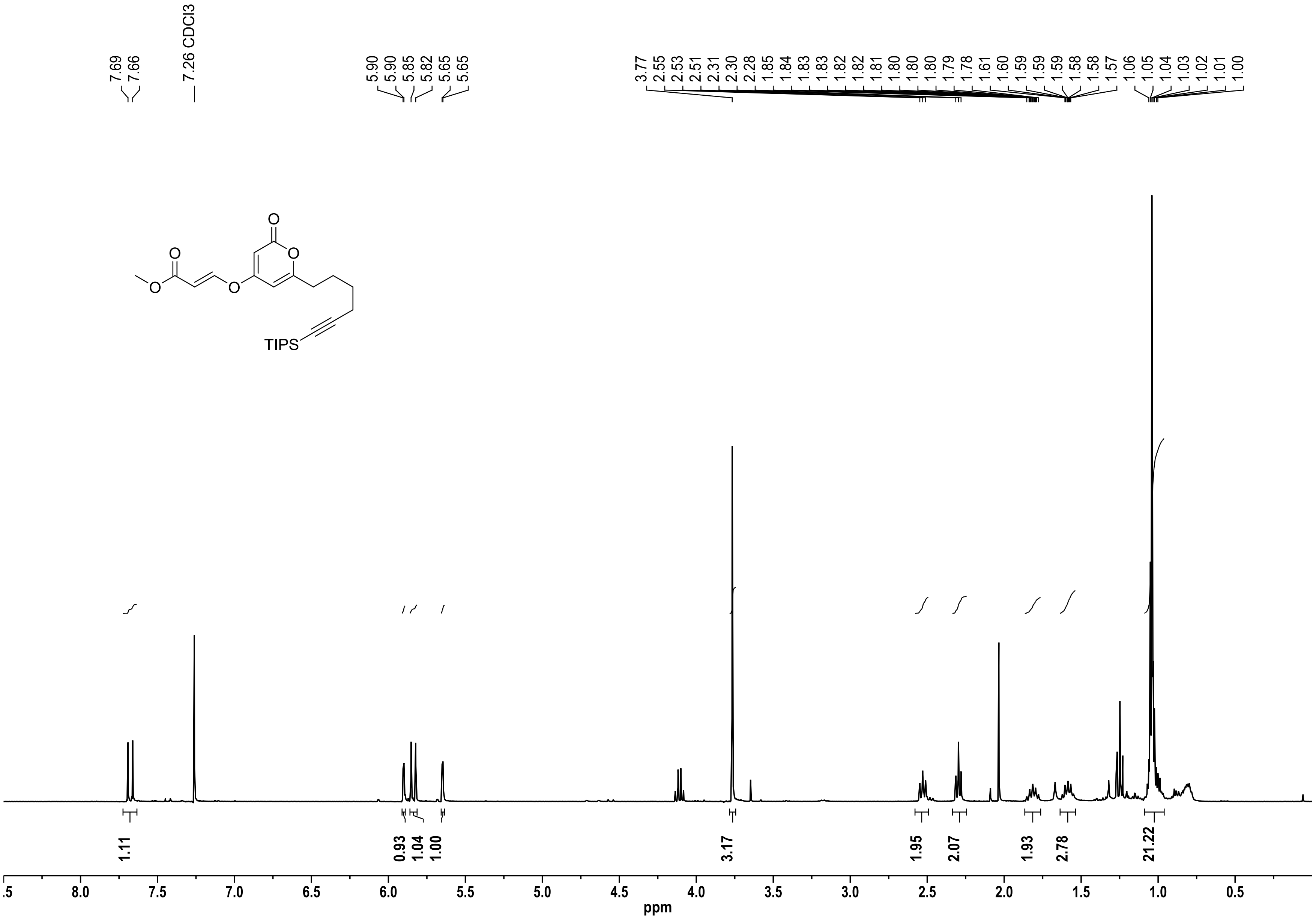

Figure S21 ^{1}H NMR spectrum of **7h**.

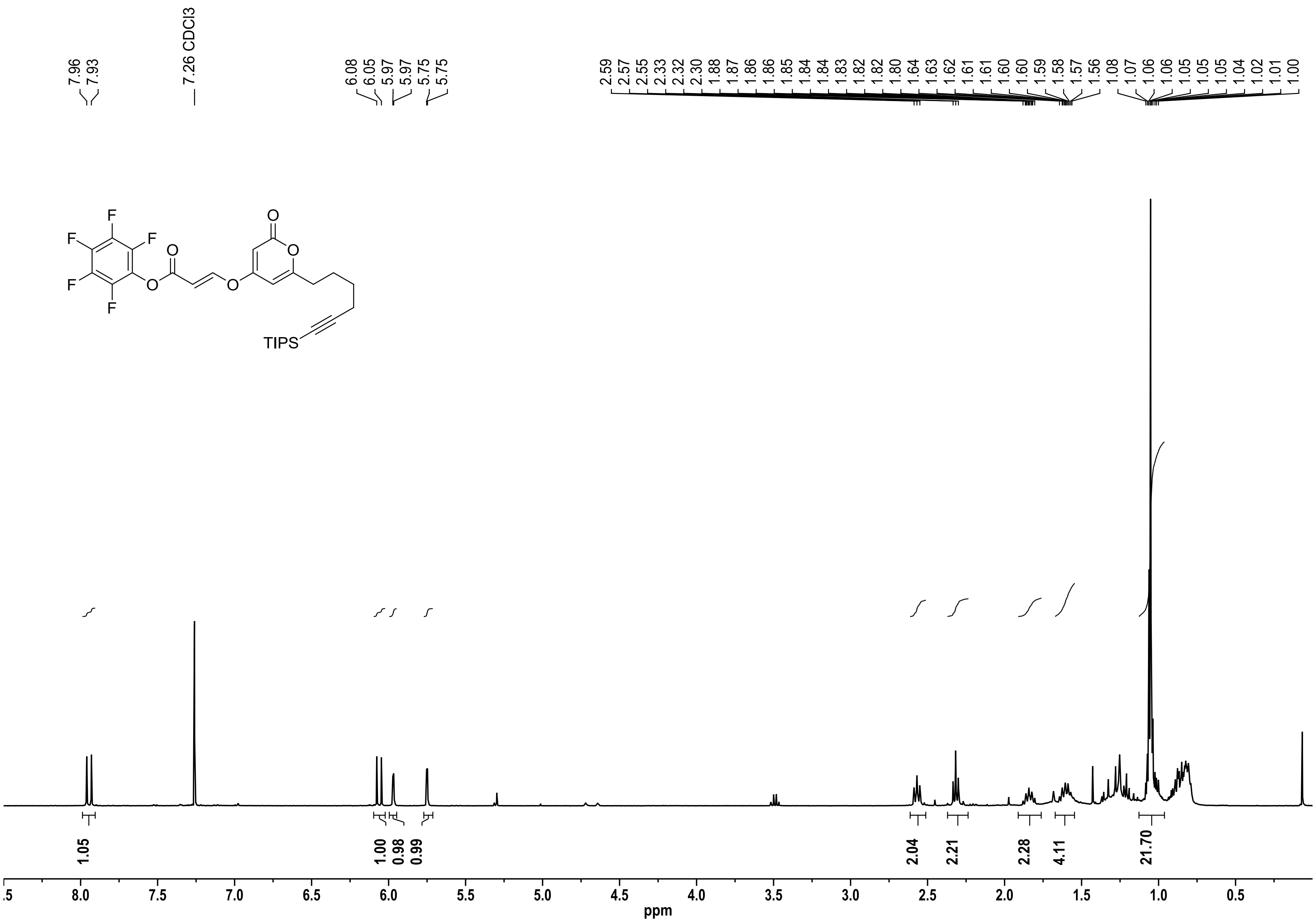

Figure S22 ^{1}H NMR spectrum of **7i**.

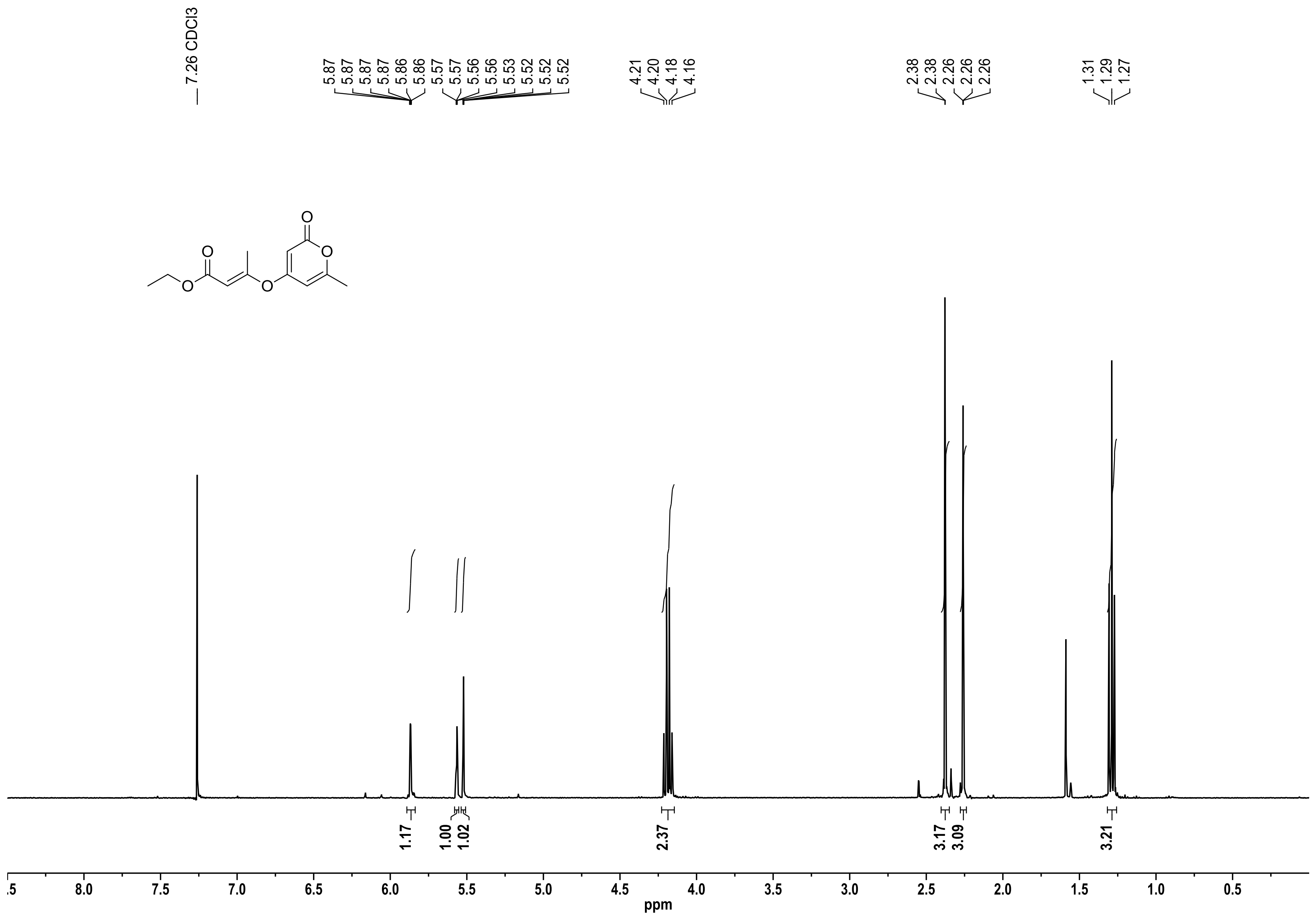

Figure S23 ^{1}H NMR spectrum of **9**.

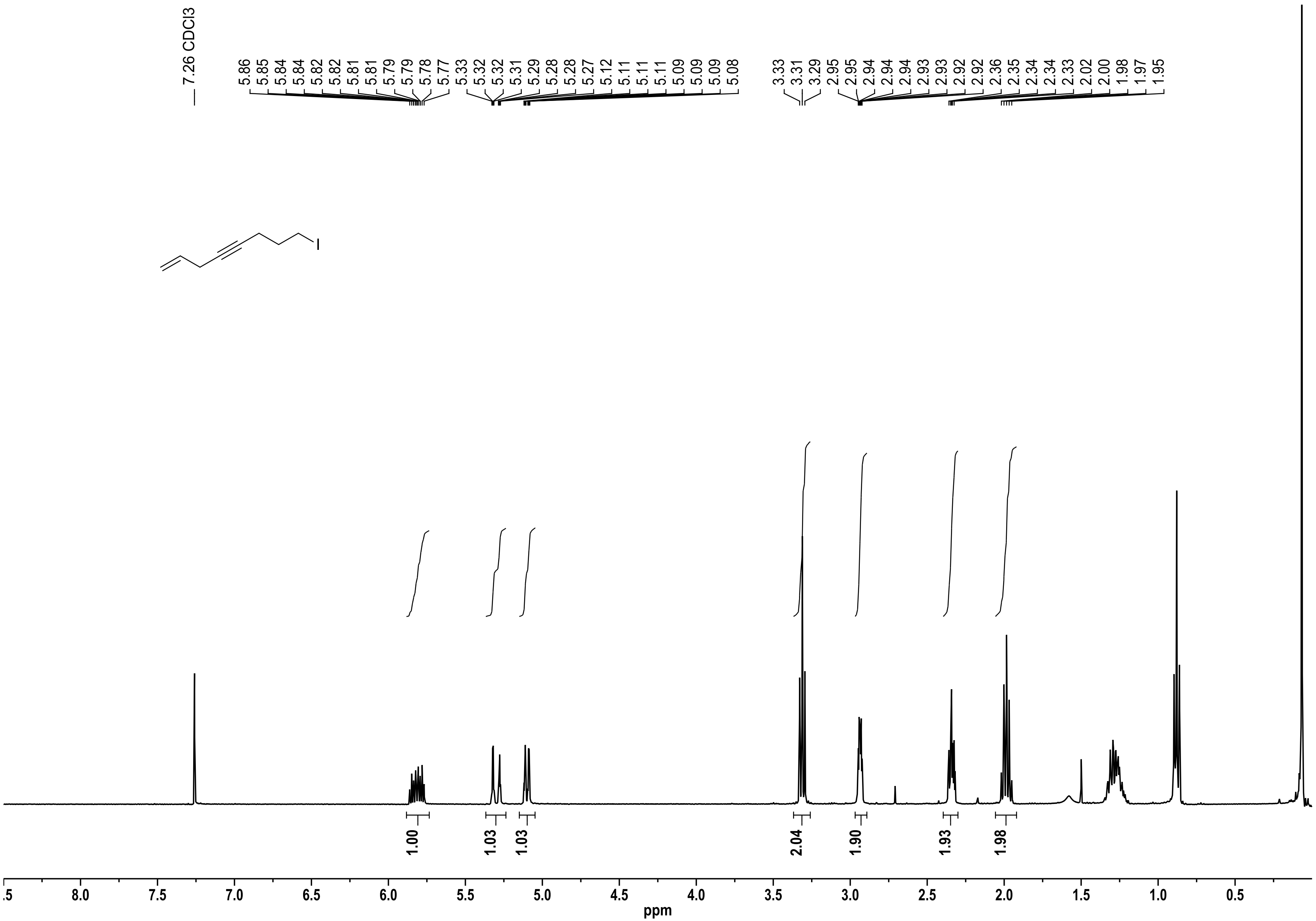

Figure S24 ^{1}H NMR spectrum of **11**.
